# Probiotic effects on brain health are both transient and sustained for several weeks after discontinuation: a randomised controlled trial

**DOI:** 10.1038/s43856-026-01800-6

**Published:** 2026-07-28

**Authors:** Ashley N. Hutchinson, Robert J. Brummer, Julia Rode

**Affiliations:** 1 School of Medical Sciences, Faculty of Medicine and Health, Örebro University, Örebro, Sweden; 2 Food and Health Centre, Örebro University, Örebro, Sweden

**Keywords:** Neuroscience, Ageing, Brain imaging

## Abstract

**Background:**

Modulating the gut-brain axis via probiotic supplementation has emerged as attractive strategy to promote brain health, but its long-term effects are largely unknown.

**Methods:**

This study with 32 community-dwelling self-reported healthy older adults (68.0 ± 5.4 years, 21 f/11 m, without contraindications for study examinations) assesses whether probiotic effects persist after intake cessation using brain imaging, targeting function (exploratory outcome of main study, primary outcome of report) and structure, and questionnaires for psychological assessments (exploratory). The 4-6 weeks discontinuation follow-up was performed after assessing immediate effects of a six-week randomised, blinded (investigators, nurses, participants), placebo-controlled parallel trial with two different formulations of *Lacticaseibacillus rhamnosus* HN001 of identical appearance/taste, in autumn 2023 in Örebro, Sweden.

**Results:**

Trial status: completed; participants analysed (of randomised) per group: *n* = 8(29) encapsulated probiotic, *n* = 12(31) non-encapsulated probiotic, *n* = 12(30) placebo; no related adverse events at discontinuation follow-up. Previously reported immediate intervention effects evoked alteration in anxiety symptom scores and distinct resting state functional connectivity patterns. While some changes persisted, others were not detectable any longer at discontinuation follow-up. Comparing both probiotic formulations, connectivity between left superior parietal lobule and right occipital/cuneal/intracalcarine/supracalcarine cortex differed significantly (seed-to-voxel analysis, T = + 7.38, cluster-size *p* = 0.0001 FDR-corrected, additionally correct for number of tests); levels for Hospital Anxiety and Depression Scale Total Score and its Anxiety Subscore differed significantly (time*group interaction effects); both between weeks 10-12 and week 6, but not baseline.

**Conclusions:**

These findings suggest that some brain-related probiotic effects may indeed persist 4-6 weeks post-intervention cessation, especially on the level of brain function as indicated by resting state functional connectivity, suggesting longer-lasting gut-brain axis effects than previously presumed.

**Registration:**

ClinicalTrials.gov NCT05801042.

## Plain language summary

Probiotics have been shown to promote overall health, including the brain. Thus far it has been assumed that probiotics need to be administered daily to exert these effects. This study asked if probiotic effects are maintained several weeks after discontinuation. To answer this question, the brain health of 32 older adults was assessed using objective brain imaging during rest and using self-administered questionnaires evaluating mood, perceived stress and sleep quality at various timepoints: before, immediately after, and 4-6 weeks after daily probiotic intake over a period of six weeks. While some changes persisted, others were not detectable any longer at follow-up. These findings suggest that probiotics may provide longer lasting effects than previously presumed, which may impact the design of future studies as well as intake recommendations.

## Abstract (web summary)

Hutchinson et al. examine if effects of probiotic supplementation on brain function persist after supplementation has been discontinued. Their findings demonstrate that some changes in resting state functional connectivity persist after intake discontinuation, while other changes in connectivity and self-rated questionnaires are no longer detected.

## Introduction

Investigation of the mechanisms underlying ageing-related diseases and healthy ageing has revealed a pivotal role for the gut-brain axis, the bidirectional route of communication between the microbiota of the gastrointestinal tract and the brain^1,2^. A growing number of studies suggests that ageing is associated with a dysbiosis of the gut microbiota, including decreased bacterial diversity^3–5^ as well as a reduction in bacterial species that produce short-chain fatty acids^6^.

Thus, modulating the gut-brain axis via probiotic supplementation has emerged as a strategy to promote healthy ageing. Several probiotic interventions in healthy older adults have shown positive effects on immune function^7–9^ and gastrointestinal symptoms^10,11^. A small number of studies have also focused on the effects of probiotic interventions on brain health in older adults, showing modest positive effects on self-reported questionnaires and cognitive test batteries^10,12^.

As only few studies have examined the effects of probiotic interventions on brain health in healthy older adults, there is a great need for more studies to understand how probiotics can impact brain function and promote healthy ageing. Until recently, all studies performed focused on self-reported rating scales and paper-pencil tests; however, the utilisation of brain imaging techniques for more objective assessment has emerged^13^. Several studies in younger participants have demonstrated that brain imaging techniques, such as magnetic resonance imaging (MRI), are sensitive enough to detect the effects of probiotic interventions on resting state brain function^14–17^. Furthermore, in our recent study, we found that a six-week intervention with encapsulated and non-encapsulated *Lacticaseibacillus rhamnosus* HN001 resulted in significant changes in brain connectivity as measured by resting state functional MRI (rsfMRI) in community-dwelling older adults (60–80 yrs)^18^.

Although increasing evidence suggests that probiotics affect brain health and function in healthy older adults, several unanswered questions remain. Importantly, although studies have demonstrated that probiotics can impact brain function, there is no strong consensus on whether these effects persist after probiotic supplementation has been discontinued. The average range of the follow-up period after discontinuation of supplementation is ~1 month, and this has been investigated in only a handful of studies^19–23^. Furthermore, although several studies utilised brain imaging assessments, such as fMRI, at baseline and immediately after the intervention, none of these studies investigated the brain imaging results after discontinuation of probiotic supplementation^22,23^. The only study to our knowledge that followed up objectively on brain function used electroencephalography and involved a 90-day intervention with a synbiotic containing *Bifidobacterium longum* W11 and fructo-oligosaccharides followed by a 30-day follow-up period once supplementation was discontinued in cirrhotic patients. They found improved cognitive performance and unchanged electroencephalography results immediately following the intervention, and these findings persisted to the 30-day follow-up period^21^.

Taken together, there is great need to understand if the effects of probiotic supplementation on brain function as assessed by imaging techniques persist after supplementation has been discontinued. To our knowledge, the present study is the first to investigate if the effects of probiotic supplementation on brain imaging, namely rsfMRI, persist or are altered after a follow-up period. To address this question, we examined the rsfMRI results and self-rated questionnaires in a subset of participants four to six weeks following our previously reported probiotic intervention^18^.

While the immediate intervention effects evoked distinct alteration in resting state functional connectivity patterns with simultaneous changes in anxiety symptom scores^18^, we demonstrate herein that some functional brain changes persist, others are not detectable any longer, and psychological symptom scores largely return to baseline levels four to six weeks after intervention end.

## Methods

### Study design

**Fig. 1:**
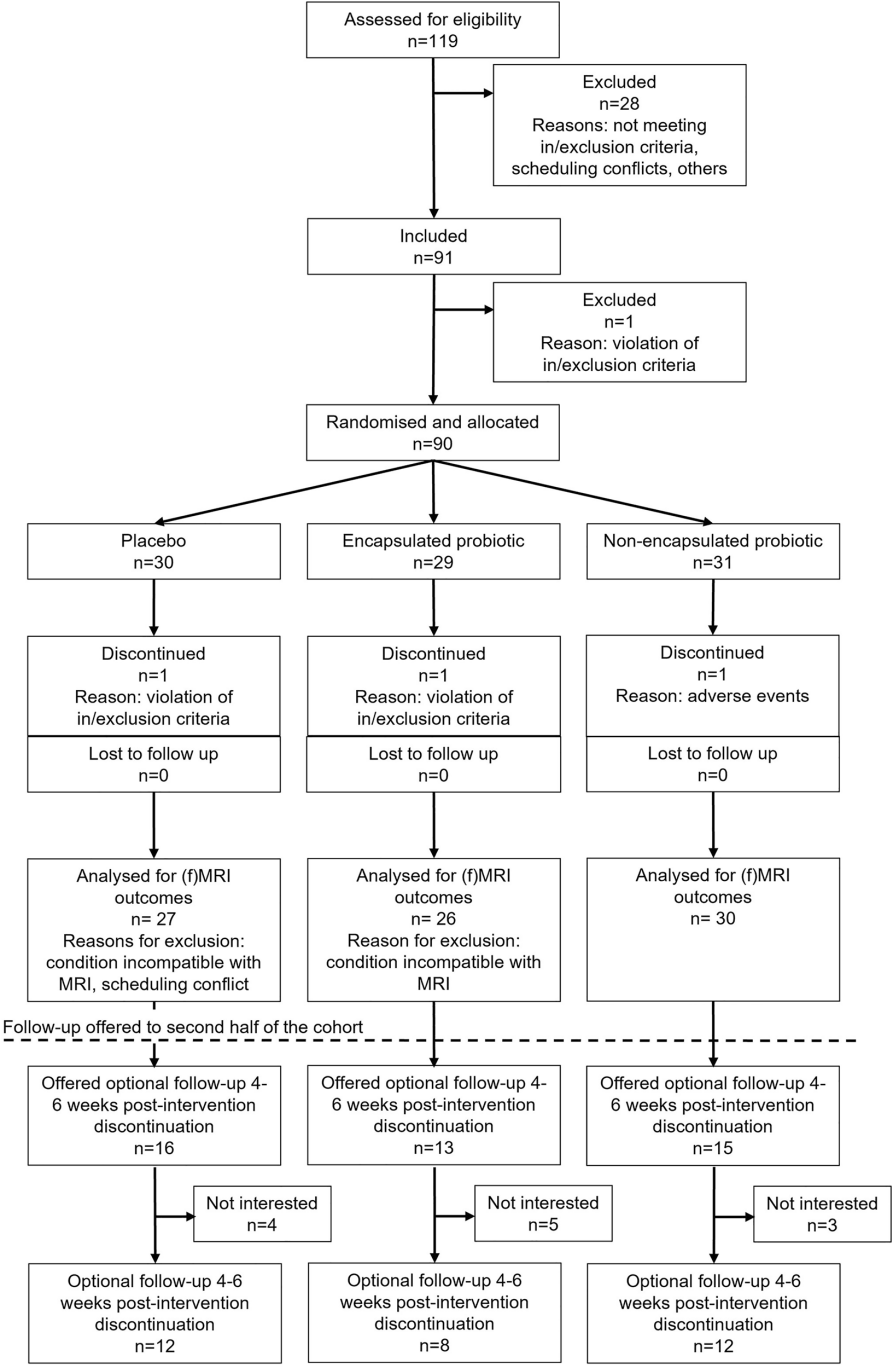
Flow chart for the participation in the optional follow-up assessment at four to six weeks post-intervention discontinuation. The original study by Rode et al.^18^ randomised a total of 90 participants and analysed 87 participants thereof for (f)MRI outcomes. From this original study, 32 participants performed the optional follow-up assessment.

A six-week randomised, double-blinded, placebo-controlled parallel study with three arms and optional visit four to six weeks post-intervention discontinuation was performed in healthy, community-dwelling, normal to overweight (body mass index, BMI: 18.5–31.9) 60–80-year-old male and female older adults (for details on the study population, please refer to Rode et al.^18^, Fig. 1 and Table 1). The aim was to evaluate the sustained effects of micro-encapsulated and non-encapsulated *Lacticaseibacillus rhamnosus* HN001 on functional connectivity as assessed by resting state functional magnetic resonance imaging (rsfMRI), grey and white matter volume as assessed by voxel-based morphometry, and psychological symptom ratings assessed by self-administered questionnaires.

**Table 1:** Study population’s background characteristics divided into groups

	*Placebo*	*Encapsulated probiotic*	*Non-encapsulated probiotic*
*n*	12	8	12
Follow-up at x days after discontinuation (mean ± SD)	32.08 ± 5.29	34.13 ± 6.27	35.17 ± 6.70
Age, years (mean ± SD, min–max)	68.8±6.1, 60-80	67.8 ± 6.3, 60–75	67.4 ± 4.1, 60–74
Sex (f/m)	8/4	6/2	7/5

Number of days, age and sex were statistically compared between the groups with either one-way ANOVA or Chi-square tests as appropriate and did not differ between intervention groups.

This is an exploratory analysis of the intervention study previously published by Rode et al.^18^. The study was performed according to the Helsinki declaration and its revisions, approved by the Swedish Ethical Review Authorities (registration numbers 2022-06042-01 for original approval and 2023-05903-02 for approval of amendment) and registered at ClinicalTrials.gov (NCT05801042, registration first posted 2023-04-06).

Participation was voluntary. Participants could withdraw at any time and for any reason without revealing the reason. Written informed consent was collected before any study-related activity. The second half of participants with full MRI datasets (from baseline and intervention end) from the original study, examined at Örebro University, Örebro, Sweden, in the autumn of 2023, were offered an optional follow-up visit (data collection 2023-11-22 to 2023-12-07). For this follow-up study, participants received an additional 300 SEK (taxable income) as compensation for time and discomfort.

For this exploratory assessment, no sample size calculation was performed. For sample size considerations, randomisation and blinding of the original study, please refer to Rode et al.^18^. The actual number of participants per group was determined by optional participation in the additional follow-up. At this point, the participants and study team were still blinded. The analyses of the herein-reported exploratory outcomes were conducted unblinded.

The 6-week intervention consisted of either a plant-based protein micro-encapsulated probiotic or a non-encapsulated probiotic containing 6 × 10^9 CFU *Lacticaseibacillus rhamnosus* HN001, or placebo; all three study products were in powder form, were blended with 5 g maltodextrin, and were provided by AnaBio Technologies.

### Study outcomes

#### Psychological assessments

Intervention effects on mood, perceived stress and sleep quality were assessed using the Hospital Anxiety and Depression Scale (HADS), Perceived Stress Scale (PSS) and Pittsburgh Sleep Quality Index (PSQI), according to previously described strategies (including handling of missing data)^18^.

#### Neuroimaging

**Fig. 2:**
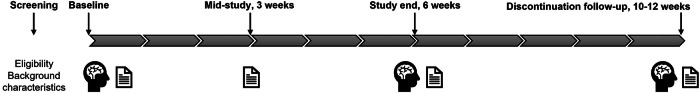
Study outline.

Functional magnetic resonance imaging (fMRI) was performed at baseline, end of intervention (week 6) and four to six weeks post-intervention (week 10-12) (Fig. 2) at the Center for Experimental and Biomedical Imaging in Örebro (CEBIO). All examinations were performed at similar times between 8 am and 1 pm, and participants were asked to keep the same morning routines, including avoidance of physical exercise and a limited consumption of caffeine to one cup of coffee or tea.

A 3.0 T MR system (Signa Premier, GE Medical Systems, WI) with both a 48-channel head coil and coils integrated within the bed was used. An initial circa 4.5-min T1-weighted structural scan (3D T1w IR-prepared fast spoiled gradient recalled echo, “BRAVO”) was performed with the following parameters applied: TR/TE = 7.3/3.0 ms, acquired voxel size 0.9 × 0.9 × 1.2 mm, parallel imaging acceleration (ARC) factor of 2. Resting state fMRI was performed for 10 min with eyes open and fixation on a cross displayed on a screen. The lights in the room were switched off. The fMRI acquisitions (gradient echo EPI pulse sequence) were performed with the following parameters applied: TR/TE = 2500/35 ms, slice thickness 2.5 mm, pixel size 2.5 × 2.5 mm, no slice gap, ARC factor of 2 and a hyperband factor of 2. Acquired images were converted to the NIFTI format using dcm2niix (https://github.com/rordenlab/dcm2niix).

### Statistics and reproducibility

#### Statistical analysis of questionnaire data and study population characteristics

Statistical tests were performed using GraphPad Prism (version 10.2.2 or 10.2.3). One-way ANOVA (number of days between intervention end and follow-up, age) or Chi-square (sex) tests were performed for study population characterisation. Two-way ANOVA and posthoc Holm-Sidak’s multiple comparisons (HADS, PSS, PSQI) were used to compare follow-up to all other timepoints or follow-up between groups. Statistical analyses were performed on original scale data. QQplots of residuals were used for visual inspection for normality and Shapiro-Wilk test was used for normality check of original data. Also, sensitivity analysis was performed (Supplementary Information 1). Although data were normally distributed, they are presented as median and interquartile range (IQR) for comparability with the original publication^18^. Posthoc tests were evaluated if ANOVA results revealed a significant time*group interaction (*p* < 0.05), and posthoc test results are presented if *p* < 0.05 uncorrected and *p* < 0.1 multiplicity corrected.

#### Resting state functional connectivity analysis

Resting state functional connectivity analysis was performed using CONN Toolbox^24^ (version 20.b, using Matlab R2020b, The Mathworks Inc., Natick, MA, USA), applying default settings if not stated otherwise, with preprocessing including standard spatial normalisation to MNI space, denoising, first- and second-level analyses as described previously^18^.

During preprocessing, on average, 2.1 volumes were removed per participant and session (of a total of 240 volumes per participant and session). The data were of high quality as assessed by visual inspection and confirmed by FC-QC association levels of *r* = 0.00 ± 0.17 and 98.2% match with non-existence of FC-QC associations for maximal global signal change, *r* = 0.00 ± 0.18 and 98.6% for maximal motion, *r* = −0.05 ± 0.18 and 88.0% for mean global signal change, *r *= 0.06 ± 0.19 and 85.9% for mean motion and *r* = −0.03 ± 0.18 and 94.2% for valid scans. As additional quality control check-point during analyses preparation, we regularly confirm that the default mode network, which is the typical task-negative network, can be picked up reliantly, to assess the validity of our fMRI acquisition and results, despite the small number of subjects per group.

The long-term intervention effects were assessed considering the four to six weeks post-discontinuation follow-up timepoint versus baseline and intervention end between groups, assessing interaction effects (contrasting two groups at a time) with respect to differential functional connectivity at resting state, independent of the result of F-tests for any difference. Based on the findings by Rode et al.^18^ that previously reported immediate intervention effects, hypothesis-driven, seed-to-voxel analyses (using parametric methods) were performed with each of the 21 a priori selected regions of interest (based on the Harvard-Oxford Atlas) as individual seeds: atlas.Hippocampus (R)/(L), atlas.Amygdala (R)/(L), atlas.AC/PC, atlas.Precuneous, atlas.p/aSMG (R)/(L), atlas.SPL (R)/(L), atlas.SFG (R)/(L), atlas.MidFG (R)/(L), atlas.IFG tri/oper (R)/(L). Results of time*group interaction effects are reported with a voxel threshold of *p* < 0.001 and a cluster threshold of *p* < 0.05 FDR-corrected. To further account for multiple seed regions and pairs of analyses, additional Bonferroni correction was applied, which led to cluster size *p* < 0.0004 (i.e. 0.05/(21 seeds × comparison of 2 pairs of timepoints × 3 intervention group pairs)) FDR-corrected. Individual connectivity values (i.e. per timepoint and participant) for time*group interactions significant after this additional multiplicity correction were exported from CONN and two-way ANOVA with posthoc pairwise comparisons, also Bonferroni corrected, conducted as well as graphs plotted in GraphPad Prism. Multiple linear regression analysis using change scores (delta value of discontinuation follow-up−immediate intervention end) of functional connectivity and psychological symptom ratings (HADS, PSQI, PSS) was also performed in GraphPad Prism.

In addition, ROI-to-ROI analyses were performed with default settings using the Harvard-Oxford atlas ROIs readily available in CONN, thus investigating 132 anatomical ROIs and 8646 connections between those, to assess whole-brain interventional effects. Moreover, a targeted ROI-to-ROI analysis was performed between all of the above-listed 21 a priori selected regions of interest. To further account for multiple ROI-to-ROI analyses, additional Bonferroni correction was applied, which led to cluster size *p* < 0.0042 (i.e. 0.05/(2 separate ROI-to-ROI analyses × comparison of 2 pairs timepoints × 3 intervention group pairs) FDR-corrected.

#### Voxel-based morphometry analysis

Analysis of the structural MRI data was performed using SPM12 (Statistical Parametric Mapping, The Wellcome Centre for Human Neuroimaging, UCL Queen Square Institute of Neurology, London, UK) in Matlab R2020b with processing as described previously^18^ and a dataset-specific DARTEL^25^ template. A full factorial design matrix with three levels for group (encapsulated probiotic, non-encapsulated probiotic, placebo) and three levels for time (baseline, intervention end, follow-up) was set up, taking the longitudinal character of the dataset into account. No implicit or explicit masking was applied. The voxel-wise level cluster-defining threshold was set to *p* < 0.001 (uncorrected) with an arbitrary cutoff at >10 voxels for cluster size. For multiplicity correction, cluster-level FDR (FDRc) correction was used (<0.05). F-contrast and T-contrasts were used to examine time*group interaction effects, time effects and group effects on grey and white matter volumes.

## Results

From the previously reported main study^18^, about 1/3 of participants were followed up four to six weeks after discontinuing the probiotic supplementation, with an overall average of 33.75 ± 6.05 days (Table 1) after discontinuation of intervention intake for this exploratory assessment. This resulted in a total of 32 (see Fig. 1 for a flow chart) community-dwelling older adults, mean age ( ± SD) 68.0( ± 5.4) years, both female (*n* = 21, 65.6%) and male (*n* = 11, 34.4%) participants, who belonged to one of the three study groups: placebo (*n* = 12), encapsulated probiotic (*n* = 8), or non-encapsulated probiotic (*n* = 12); and whose data was included in all herein presented analyses. Age and sex distribution did not differ between the groups (Table 1). For further details on phenotypic characteristics, including comorbid diseases or concomitant medications, as well as compliance and adverse events occurring during the intervention, please refer to Rode et al.^18^.

### Mood, perceived stress and sleep

**Fig. 3:**
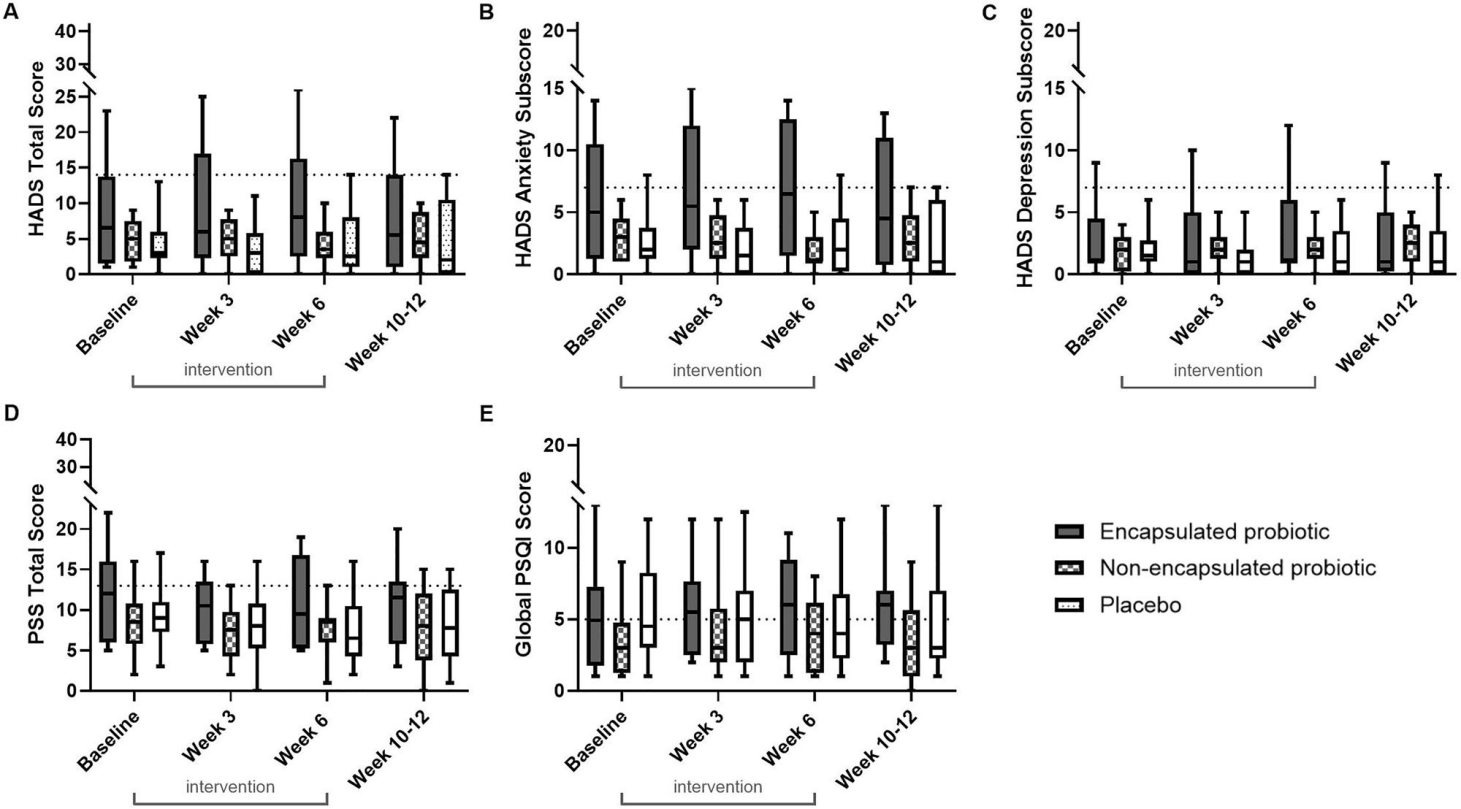
Intervention effects on mood, perceived stress and sleep quality. **A** The Hospital Anxiety and Depression Scale (HADS), **B** its Anxiety and **C** its Depression Subscore. **D** the Perceived Stress Scale (PSS) and **E** the Pittsburgh Sleep Quality Inventory (PSQI). For all, a lower score is better. The dotted line indicates the clinical cutoffs. Data are visualised as boxplots, line presents median, box presents interquartile range, and whiskers present minimum to maximum. White box filling represents the placebo, grey the encapsulated probiotic, and grey-white checkerboard pattern the non-encapsulated probiotic intervention groups. Statistical comparisons by two-way ANOVA and posthoc Holm-Sidak’s multiple comparisons, two-sided, with *n* = 32 human participants.

On the Hospital Anxiety and Depression Scale (HADS) Total Score, a significant time*group interaction effect (*F*(6,87) = 2.299, *p* = 0.0416) was observed (Fig. 3A). Posthoc multiple comparisons revealed that this originated from a decrease from intervention end (week 6) to discontinuation follow-up (week 10-12) (mean diff = −2.00, SE diff = 0.5669, *t *= 3.528, DF = 7, *p* = 0.0096, adjusted *p *= 0.0286) in the encapsulated probiotic group. Visual inspection further indicates that scores in the encapsulated probiotic group appear to increase from baseline until intervention end and thereafter decrease back to baseline levels at follow-up.

On the HADS Anxiety Subscore, a significant time*group interaction effect (*F*(6,87) = 2.696, *p* = 0.0190) and a significant main group effect (*F*(2,29) = 3.786, *p* = 0.0346) were observed (Fig. 3B). Here, in the encapsulated probiotic group, scores decreased non-significantly from intervention end (week 6) to discontinuation follow-up (week 10-12) (mean diff = −1.250, SE diff = 0.4532, *t* = 2.758, DF = 7, *p* = 0.0282, adjusted *p* = 0.0821). Visual inspection further indicates that scores in the encapsulated probiotic group appear to increase from baseline until intervention end and thereafter decrease back to baseline levels at follow-up. A vice versa pattern with decrease until intervention end and (non-significant) increase back to baseline levels at follow-up is visual in the non-encapsulated probiotic group.

On the Perceived Stress Scale (PSS), a significant main time effect (*F*(2.871,83.25) = 3.141, *p* = 0.0314,) was observed (Fig. 3D). No posthoc multiple comparisons are reported, since no significant time*group interaction was observed.

At follow-up, there was no significant difference between the intervention groups on any of the scores.

Furthermore, the HADS Depression Subscore (Fig. 3C) and scores on Pittsburgh Sleep Quality Inventory (PSQI; Fig. 3E) were not significantly affected.

The two-way ANOVA revealed highly significant between-subject effects for all scores and subscores (*p* < 0.0001).

For additional information, including baseline comparisons, please refer to Supplementary Information 1 and Supplementary Table 1.

### Resting state functional connectivity

**Fig. 4:**
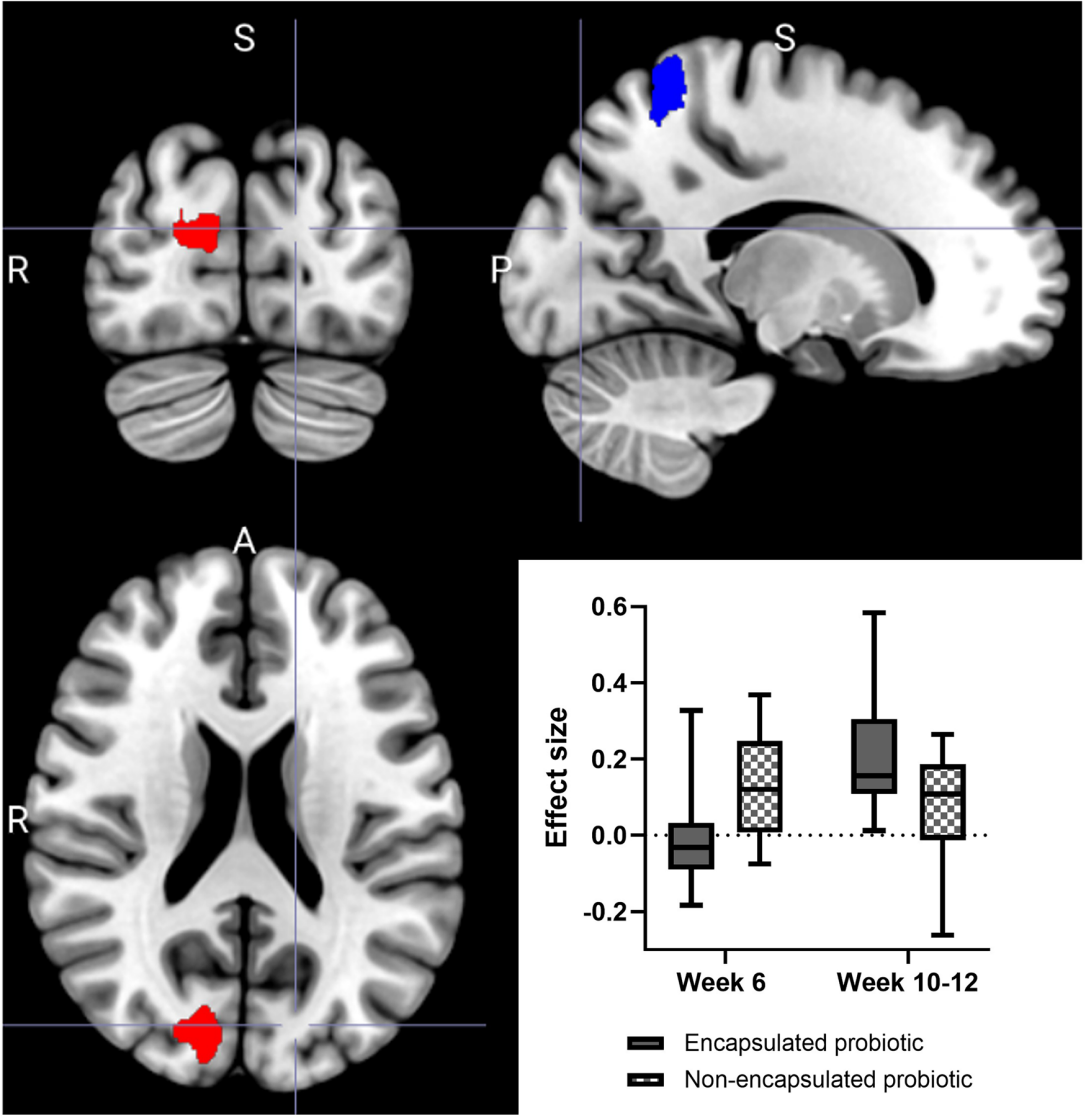
Resting state functional connectivity at discontinuation follow-up (week 10–12) versus intervention end (week 6). Seed-to-voxel analyses (second-level mixed effects analyses, using a full factorial general linear model, two-sided) of 21 a priori selected regions of interest revealed, for the comparison of both probiotic formulations, altered connectivity between the seed region superior parietal lobule left (blue) and the target cluster (red) covering occipital pole, lateral occipital cortex superior division, cuneal cortex, intracalcarine cortex, and supracalcarine cortex, all in the right hemisphere (cluster-size *p* < 0.05/(21 × 2 × 3) FDR-corrected and voxel threshold *p* < 0.001 uncorrected). Seed region and target cluster are visualised superimposed on a standard brain template in MNI space, visualised in the coronal, sagittal and axial plane. The graph depicts the connectivity strength across all sessions/participants denoted as effect size of the correlation (as boxplots, line presents median, box presents interquartile range, and whiskers present minimum to maximum; grey box filling represents the encapsulated probiotic, and grey-white checkerboard pattern the non-encapsulated probiotic intervention groups). Statistical comparisons by two-way ANOVA and posthoc two-sided t-tests, Bonferroni corrected, with *n* = 32 human participants.

The intervention effects observed by Rode et al.^18^ as assessed by hypothesis-driven seed-to-voxel analyses, using each of the 21 a priori selected regions of interest as seeds, were further investigated. Synchronicity of the seed regions’ activity with any other region (denoted as cluster, i.e., a number of neighbouring voxels) of the brain can be described as correlations and anti-correlations. Both probiotic interventions affected resting state functional connectivity in the comparison of discontinuation follow-up with baseline (Supplementary Table 2) and immediate intervention end (Supplementary Table 3), although the intervention-related differences varied depending on both the intervention group comparison as well as the timepoint in question. In the comparison from follow-up (week 10–12) to baseline, the majority of intervention-related alterations in functional connectivity (for all comparisons) were changes in anti-correlation (7/9) (Supplementary Table 2), whereas the majority of intervention-related alterations of functional connectivity when comparing follow-up (week 10–12) to intervention end (week 6) were changes in correlation (9/10) (Supplementary Table 3). Also, the seed regions involved in those two comparisons differed (Supplementary Table 2 versus Supplementary Table 3). One of those effects remained significant (*p* < 0.0004) after additional multiplicity correction for the number of seeds tested, comparisons of two pairs of timepoints and of three intervention groups, and is presented in Table 2 and Fig. 4.

**Table 2:** Resting state functional connectivity at discontinuation follow-up (week 10–12) versus intervention end (week 6)

Group comparison	A priori selected seed region	Target cluster size [number of voxels]	Target cluster-size p-FDR corrected	Target cluster peak coordinate [xx yy zz]	Target cluster coverage (order of anatomical regions based on proportion of coverage from largest to smallest)	Intervention-related alterations of connectivity [T]	Intervention-related alterations of connectivity^a^ [description]
Placebo vs Encapsulated probiotic	--- none ---						
Placebo vs Non-encapsulated probiotic	--- none ---						
Encapsulated probiotic vs Non-encapsulated probiotic	Superior Parietal Lobule Left	379	0.0001	+20 −96 +30	Occipital Pole Right, Lateral Occipital Cortex superior division Right, Cuneal Cortex Right, Intracalcarine Cortex Right, Supracalcarine Cortex Right	+7.38	• correlation decreased upon discontinuation of the non-encapsulated probiotic • correlation increased upon discontinuation of the encapsulated probiotic

Significant time*group interactions; all voxel threshold *p *< 0.001 uncorrected and cluster threshold *p* < 0.05 with exact *p*-value as indicated (hypothesis-driven seed-to-voxel analysis (second-level mixed effects analyses, using a full factorial general linear model, two-sided), regions annotation based on Harvard-Oxford atlas). Table only reports alterations that remain significant, when additional multiplicity correction is applied for the number of seeds tested, comparisons of two pairs of timepoints and of three intervention groups (cluster size *p* < 0.0004 FDR-corrected).

^a^Synchronised brain activity patterns are described as correlations (e.g. two brain regions showing the same activity pattern) and anti-correlations (e.g. two brain regions showing opposite activity patterns).

Namely, when considering the comparison of discontinuation follow-up (week 10–12) and immediate intervention end (week 6), and comparing both probiotic formulations head-to-head, functional connectivity differences were observed between the seed region superior parietal lobule left and a target cluster covering occipital pole, lateral occipital cortex superior division, cuneal cortex, intracalcarine cortex, and supracalcarine cortex, all in the right hemisphere, a significant time*group interaction effect (*F*(1,18) = 54.53, *p* < 0.0001), and a significant main time effect (*F*(1,18) = 19.32, *p* = 0.0003) was observed. Posthoc multiple comparisons (by Bonferroni correction significant at *p* < 0.0125) revealed that this originated mainly from an increase from intervention end (week 6) to discontinuation follow-up (week 10-12) (mean diff = 0.2229, SE diff = 0.02931, *t *= 7.604, DF = 18, descriptive *p* < 0.0001) in the encapsulated probiotic group. The non-encapsulated probiotic group presented a non-significant decrease from intervention end to discontinuation follow-up (mean diff = −0.05654, SE diff = 0.02393, *t* = 2.36, DF = 18, descriptive *p* = 0.0296). The two probiotic groups did not differ significantly at intervention end nor discontinuation follow-up. For this connection, an *F*-test for any difference between intervention groups was significant (*F*(2,29) = 22.07, *p* = 0.0464 (target cluster size FDR-corrected)) between the identical seed region superior parietal lobule and a largely overlapping target cluster (number of voxels = 125, peak coordinate = +14 −82 +20, coverage: occipital pole and cuneal cortex right). Multiple linear regression showed that group was the significant predictor of this functional connectivity change (*β* = 0.3188, SE = 0.05436, *t* = 5.866, *F*(1,15) = 34.40, *p* < 0.0001). When excluding the group, HADS Total Score was the significant predictor (*β* = −0.03779, SE = 0.01586, *t *= 2.383, *F*(1,16) = 5.678, *p* = 0.0299), PSS and PSQI were not significant. HADS Anxiety and Depression Subscores have not been tested due to being derived from the HADS Total Score.

When considering the comparison from discontinuation follow-up (week 10–12) to baseline, it is noteworthy that none of the functional connectivity differences remained significant after additional multiplicity correction for the number of seeds tested, comparisons of two pairs of timepoints and of three intervention groups.

No intervention-related time*group interaction effects were observed in ROI-to-ROI analyses between the 21 a priori defined regions of interest. ROI-to-ROI analyses, including all 132 atlas regions revealed significant connectivity alterations between cerebellar regions and bilateral planum temporale and polare, as well as anterior and posterior superior temporal gyrus differences in the comparison encapsulated probiotic versus placebo at discontinuation follow-up (week 10–12) versus baseline (*F*(2,17) = 19.39, p-FDR = 0.0144), but no other differences. However, this network, does not remain significant after additional multiplicity correction for two ROI-to-ROI analyses performed and comparisons of two pairs of timepoints and of three intervention groups (Bonferroni-corrected *p* < 0.0042).

### Structural brain assessment

Simultaneous to functional changes, non-significant indications of grey matter volume changes had been observed at immediate intervention end, with the largest cluster in left hippocampus and amygdala after the encapsulated probiotic and in left frontal pole for the non-encapsulated probiotic (without any differences between both probiotic formulations), as reported by Rode et al.^18^. Herein, we investigated whether those grey matter changes increase, whether other changes occur, or whether grey and white matter volume remain unaffected, upon intake cessation. At discontinuation follow-up, no time*group interaction or time effects for grey or white matter volume assessed by voxel-based morphometry were observed (*p* < 0.001 uncorrected). Significant (FDRc < 0.001) group effects were observed. Posthoc analyses revealed no suprathreshold within-group differences at follow-up versus baseline or intervention end, and no between-group differences at follow-up (*p* < 0.001 uncorrected).

## Discussion

This exploratory proof-of-concept study is, to the best of our knowledge, the first to assess probiotic effects after discontinuation of daily intake on brain health using self-administered questionnaires for psychological assessments and imaging, targeting both structure and function, of the brain. The herein reported four to six weeks post-intervention discontinuation follow-up was performed subsequently after assessing immediate intervention effects that have been reported previously^18^. The comparison of the immediate intervention effects with the discontinuation effects allows for an evaluation of transient versus sustained brain-related effects of probiotics.

The immediate intervention effects evoked distinct alteration in resting state functional connectivity patterns with simultaneous changes in anxiety symptom scores^18^. Probiotic discontinuation effects were assessed in a subset of approximately one-third of the original study population, hence, the interpretation of “no effects” should be particularly considered with caution.

Here, we report that psychological symptom scores, after having reached a visual peak, largely return to baseline levels four to six weeks after intervention end. Levels of the Hospital Anxiety and Depression Scale (HADS) Total Score and its Anxiety Subscore, respectively, differ significantly between the discontinuation follow-up and immediate intervention end but not baseline, indicating that the scores return to baseline. Perceived stress and sleep quality ratings do not show any particular trend, which is not surprising since no immediate intervention effects were observed either^18^.

While we demonstrate that some functional brain changes seem to persist for weeks after intake cessation, others are not detectable any longer; additionally, newly detected ones, so-called legacy effects, at discontinuation follow-up are observed.

Interestingly, when comparing resting state functional connectivity at the discontinuation follow-up with baseline, many seed regions and where they are projecting to show similarities to those reported at immediate intervention end^18^. As such, before additional multiplicity correction, in the comparison of the encapsulated probiotic with placebo, connectivity of the frontal gyrus, which is part of the Default Mode Network, is altered, although target clusters differ essentially when assessing discontinuation effects here, as opposed to the previously reported immediate intervention effects. Also, in the comparison of the non-encapsulated probiotic with placebo, before additional multiplicity correction, connectivity of seeds in the hippocampus or amygdala with several target clusters, similar yet smaller than those at immediate intervention end, is altered, although different hemispheres may be involved. When comparing the two probiotic formulations at discontinuation follow-up, less functional connections seem to be involved than at immediate intervention end, before additional multiplicity correction.

Furthermore, when comparing resting state functional connectivity at the immediate intervention end with discontinuation follow-up, before additional multiplicity correction, the comparison of the non-encapsulated probiotic with placebo shows similarities to those versus baseline, while the comparison of the encapsulated probiotic with placebo—which yielded the least robust results at immediate intervention end—did not reveal any significant differences. Noteworthy, the comparison of both probiotic formulations still revealed functional connectivity differences, the target cluster of which, although a different seed, showed very similar coverage and directionality to the one altered between immediate intervention end and baseline^18^. Interestingly, this connectivity alteration is the only one in the herein presented work, and the only one of the previously reported^18^ immediate intervention effects, that remain statistically significant even after additional more strict multiplicity correction for the number of seeds tested. Other probiotic intervention studies also observed altered resting state functional connectivity involving the superior parietal lobule, intracalcarine and supracalcarine cortex, and occipital pole and cortex, but not the cuneal cortex^14,15,17^.

Noteworthy, hypothesis-free whole-brain ROI-to-ROI analyses revealed significant alterations that were not detected at immediate intervention end. However, those do not remain significant after additional multiplicity correction.

When interpreting the involvement of the various seed regions and the coverage of the target clusters, it is important to note that the Harvard–Oxford Atlas is a probabilistic atlas^26^ and that connections may still be involved in similar processes; even though, brain regions are annotated slightly differently, e.g. by closely neighbouring regions.

When considering the comparison from discontinuation follow-up (week 10–12) to baseline by intervention groups, it is to note that the non-significant (after additional multiplicity correction) functional connectivity differences in the encapsulated probiotic group involved regions associated with visual processing, whereas the non-significant differences in the non-encapsulated probiotic involved regions associated with visual and language processing, emotional regulation as well as memory, both compared to placebo. When considering the comparison of discontinuation follow-up and immediate intervention end (week 6), the non-significant functional connectivity differences in the non-encapsulated probiotic group also involved regions associated with visual and language processing and emotional regulation, and furthermore episodic memory and attention, compared to placebo, while the encapsulated probiotic did not differ from placebo. A head-to-head comparison of both probiotic formulations for discontinuation follow-up versus baseline revealed non-significant differences in functional connectivity involving regions associated with language processing, attention, episodic memory, and spatial processing, while for discontinuation versus immediate intervention end significant functional connectivity differences were observed in regions associated with spatial perception, attention, visual processing, working memory. In terms of connectivity across functional domains in the present study, the differences between the two probiotic formulations largely resemble the observations at immediate intervention end, as previously reported^18^.

So far, it is not possible to conclude whether one formulation may be superior to the other. Yet, it is to speculate that the variations in effects may be due to different modes of actions of the two formulations. Possibly, the encapsulated probiotic reaches more distal parts of the gastrointestinal tract in higher live/dead ratios, than the non-encapsulated probiotic does, as highlighted in Rode et al.^18^. Our recent systematic review showed that most of the resting state functional magnetic resonance imaging (rsfMRI) studies assessing probiotic effects use powdered products, presumably with non-microencapsulated formulations^13^.

The variation of transient and persistent effects of the probiotic intervention on brain function might be explainable by a mixture of i) gradual adaptive responses to return to baseline, and ii) gradual adaptive responses not yet detectable at immediate intervention end (due to their weak or scattered nature) but detectable after additional weeks.

A recent meta-analysis and meta-regression of few probiotic studies assessing depressive symptoms showed that the evaluation timepoint, whether immediately after intervention end or after a given discontinuation period, did not affect the overall intervention effect result, while effect sizes could be affected^27^.

Careful consideration of the individual evidence reveals that probiotic follow-up effects on brain health, however, remain inconclusive. Some studies suggest that the probiotic effects might persist beyond the intervention period, while others suggest that regular continued intake is potentially necessary for sustained effects. The following extracts describe the diverse landscape of the research on probiotics’ transient versus sustained effects^19–23,28–31^.

In patients with a depression diagnosis but without antidepressant medications, an eight-week intervention with Ecologic Barrier showed that scores on several questionnaires evaluating depression, anxiety and stress remained decreased at one-month follow-up^20^. There, from baseline to study end and then to one-month follow-up, participants were more likely to move from a subclinical diagnosis of depression to no depression diagnosis in the probiotic group, resulting in a change from clinical to subclinical as the median rank category^20^. A significant time effect was observed in the probiotic group only, with differences in scores at follow-up compared to baseline as well as study end, but not between study end and baseline^20^. No significant between-group differences at follow-up versus study end scores were observed^20^.

In patients with a depression diagnosis and on active antidepressant medication, a 31-day intervention with Vivomixx, a probiotic mixture containing several Bifidobacteria and Lactobacilli and a *Streptococcus thermophilus*, caused depression scores from baseline to decrease more in the probiotic group compared to placebo four weeks post-intervention, while the scores were visually similar at study end and follow-up together with simultaneous effects on gut microbiota composition in terms of individual taxa and diversity, generally^23^. In the same study, functional magnetic resonance imaging (fMRI) was utilised to capture brain-level probiotic effects at baseline and immediate intervention end, but those measurements were not repeated at follow-up^23^.

In irritable bowel syndrome patients, a six-week intervention with *Bifidobacterium longum* NCC3001 compared to placebo resulted in a reduction of the number of participants with high depression scores, an effect that was sustained also four weeks post-discontinuation^22^. At immediate intervention end, measures of quality of life also showed improvement. Visually, some scores remained improved, while others worsened again although not reaching baseline levels. However, for most scores, no between-group differences could be reported at follow-up^22^. This study also performed fMRI at baseline and intervention end, but did not repeat such measure at follow-up.

One previous study indeed performed objective measurements of brain function using electroencephalography before, immediately after and 30 days after intervention end. There, in cirrhotic patients, 90-day intake of a synbiotic Zirfos containing *Bifidobacterium longum* W11 and fructo-oligosaccharides resulted in improved cognitive performance but not electroencephalography measures over time and compared to the placebo group, both at immediate intervention end and after additional 30 days of follow-up^21^.

While the above-mentioned studies suggest that four weeks of discontinuation follow-up seem the most commonly investigated in terms of probiotics’ long-term effects on the gut–brain axis, even shorter as well as longer follow-up periods have been evaluated previously^28,29,31^.

As such, in patients with a sub-threshold depression, a twelve-week intervention with Bifizen, a multi-strain probiotic mixture containing amongst others *Lacticaseibacillus rhamnosus* LR06, resulted in sustained decreased scores of several questionnaires evaluating depression symptoms six weeks after intervention cessation, while decreased scores on several questionnaires evaluating anxiety symptoms at immediate intervention end were transient^31^. A six-week intervention with the same probiotic mixture in healthy subjects resulted in improved subjective sleep quality (PSQI global score and sleep latency subscore), but had no effects on objective sleep measures, both at immediate intervention end and after an additional three-week follow-up period^30^. In both studies, solely within-group effects but no between-group or interaction effects were observed^30,31^. A study with one- and two-week post-intervention discontinuation measures of perceived stress indicated that scores almost immediately, although somewhat gradually, returned to baseline^32^.

The interpretation of the herein reported baseline versus discontinuation follow-up (week 10–12) and immediate intervention end (week 6) versus discontinuation follow-up (week 10–12) results is limited by a comparison to previously assessed baseline versus immediate intervention end (week 6) effects in a larger study population. We have chosen not to reassess immediate intervention effects, other than presenting Supplementary Table 1, as this would have been a republication of the same data in an arbitrary subset of participants not meeting the pre-set study criteria. Also, the sample size for the reported analyses is rather small, which limits interpretability, yet it is acceptable for the proof-of-concept nature of this work. Generally, the effects seem meaningful since they are detectable even in this small sample size. Other studies were able to show immediate effects of probiotic interventions in similar sized groups in homogenous healthy^16^ as well as in more heterogenous compromised populations^23^. Phenotypic characteristics at baseline, including ratings on psychological symptom scores (HADS, PSS, PSQI), were evenly distributed across groups. To note is that the herein investigated study population was healthy, community-dwelling older adults that on average presented normal (i.e. below clinical cutoffs) psychological symptom scores (e.g. on HADS) with limited room for improvement, which may have led to some outcomes encountering ceiling effects. Also, within-group variability tended to be larger than between-group differences. With regard to the hypothesis-generating character of the performed study, opposed to an accurate description of sustained intervention effects, effects were reported based on liberal significance thresholds. Multiplicity correction was performed and reported next to descriptive *p*-values for the questionnaire data. For the fMRI data, cluster-size FDR-correction for reporting of effects was performed at the standard threshold of <0.05. Exact *p*-values are given per altered connectivity, enabling a stricter interpretation by the reader, using for example additional Bonferroni correction based on the number of seeds tested, comparison of pairs of timepoints and pairs of intervention groups, as indicated, if wished for. Sustained intervention effects were assessed with respect to baseline as well as immediate intervention end timepoints, for the same reason of hypothesis generation. The analyses of questionnaire data (HADS, PSS, PSQI) were primarily performed by two-way ANOVA since this allowed the full utilisation of the present study design. We acknowledge that the combination of small sample size, zero-bounded and skewed distribution of scores, as well as potential ceiling effects may, however, violate the assumption of general robustness to moderate deviations of residuals from normal distribution. Sensitivity analyses with non-parametric tests (Supplementary Information 1) yielded results consistent with the presented parametric analyses, supporting the robustness of effects under different distributional assumptions.

Due to the fact that the additional follow-up was optional for participants and at a stage where the study was still blinded, the comparison groups were not equally sized, and length of follow-up period became varied by random factors such as scheduling options. Yet, the ongoing blinding during data collection is a direct strength of this study. The exploratory data analyses, however, were performed unblinded but according to pre-defined standards, due to priority given to the primary and secondary outcomes of the original study. The second half of the original study population was offered to continue participation in this optional follow-up investigation. This resulted in a final subset of approximately a third of participants of the original study population. Another perceived limitation may be that probiotic effects on the gut microbiota itself were not assessed in this study. We have, however, previously shown that systemic, including gut-brain axis, physiological effects are possible without substantial alterations of faecal microbiota composition^33^. Furthermore, it is unclear whether probiotic interventions may affect gut microbiota composition^34^ and whether such effects may be persistent.

Generally, the analyses of discontinuation effects were restricted to psychological symptom scores as well as structural and functional brain changes. The grey matter volume effects of the immediate intervention were not very robust^18^, thus it is not surprising that those effects were not sustained. However, these findings may suggest that brain structure seems to be quite plastic when targeted by gut-brain axis modulating interventions.

Immediate intervention effects on self-administered questionnaires for assessment of psychological symptoms were rather subtle^18^, which could have led to difficulties in evaluating changes after an additional follow-up period. Mood alterations in particular seemed to return to baseline levels, an observation supported both statistically and visually. Similar, yet more difficult to interpret, transient effects combined with sustained and newly observable alterations on resting state functional connectivity were seen. Such phenomena are especially important to consider when planning cross-over designed studies, where the typical four-week washout period might not suffice for resetting effects of brain function, at least not for the herein tested probiotic. Future larger studies should even include multimodal integrative analyses.

Together, our results indicate that some brain-related probiotic effects of just six-weeks of active intervention may be longer lasting than originally expected, highlighting their potential for mental well-being, here in an elderly population. These findings support the need to investigate the mechanisms underlying the persistent effects of probiotics on brain function.

## Supplementary information

Transparent Peer Review file

Supplementary Information

Description of Additional Supplementary Files

Supplementary Data File

## Data Availability

The data are pseudonymised, and the key variable may not be destroyed. According to Swedish ethics regulations, the raw data cannot be shared without an approved ethics application from the Swedish National Ethics Authority. An ethical permit can only be obtained for research being conducted within Sweden. Data can be requested as a public document via forskningsdata@oru.se and will have to undergo a confidentiality assessment to assess what can be released. Numerical results underlying the graphs presented in Figs. 3 and 4 are found in the Supplementary Data File.
