# Supplementary material for: Probiotic effects on brain health are both transient and sustained for several weeks after discontinuation: a randomised controlled trial
Source: Commun Med (Lond). 2026 Jul 28;6:418. doi: 10.1038/s43856-026-01800-6 (PMC13415881; doi:10.1038/s43856-026-01800-6)
Supplement: Supplementary file 2 — Supplementary Information [file 43856_2026_1800_MOESM2_ESM.pdf]

SUPPLEMENTARY INFORMATION

Probiotic effects on brain health are both transient and sustained for several weeks after discontinuation: a randomised controlled trial

Ashley N Hutchinson[1,2], Robert J Brummer[1,2], Julia Rode*[1,2]

[1]School of Medical Sciences, Faculty of Medicine and Health, Örebro University, Örebro, Sweden

[2]Food and Health Centre, Örebro University, Örebro, Sweden

*Correspondence: Julia Rode, School of Medical Sciences, Faculty of Medicine and Health, Örebro University, Örebro, Sweden, email: julia.rode@oru.se, phone: 0046 19 30 3000

Supplementary Information 1. Sensitivity analysis.

To evaluate robustness of the two-way ANOVA analyses of psychological symptom ratings (HADS Total Score, HADS Anxiety Subscore, HADS Depression Subscore, PSS, PSQI), non-parametric Friedman tests were assessed per group (encapsulated probiotic, non-encapsulated probiotic, placebo) with *posthoc* Dunn's multiple comparisons comparing discontinuation follow-up with every other timepoint, and non-parametric Kruskal-Wallis tests were performed between groups with *posthoc* Dunn's multiple comparisons at discontinuation follow-up (week 10-12). Friedman, Kruskal-Wallis, and Dunn's test results are presented if p<0.1.

HADS Total Score within the encapsulated probiotic group between timepoints differed near-significantly (Friedman statistic=7.500, p=0.0576), *posthoc* Dunn's multiple comparison test revealed that this difference originated from a decrease from intervention end to discontinuation follow-up (p=0.0118, adjusted p=0.0355).

HADS Anxiety Subscore within the encapsulated probiotic group between timepoints differed near-significantly (Friedman statistics=7.456, p=0.0587), *posthoc* Dunn's multiple comparison tests revealed that this difference originated from a decrease from intervention end to discontinuation follow-up (p=0.0201, adjusted p=0.0604).

HADS Anxiety Subscore within the non-encapsulated probiotic group between timepoints differed near-significantly (Friedman statistics=7.780, p=0.0508), *posthoc* Dunn's multiple comparison tests were not significant.

No other within-group (i.e. between-timepoint) differences were observed for any other group or score. Neither were any between-group differences observed at discontinuation follow-up.

Those results are consistent with the results observed by two-way ANOVA and *posthoc* Holm-Sidak's multiple comparisons as reported in the main text and Figure 4, supporting their robustness.

__Supplementary Table 1.__ Psychological symptom ratings compared between all timepoint and intervention groups in this sub-population (n=32), no multiplicity correction applied.

	**HADS total**			**HADS anxiety**			**HADS depression**			**PSS**			**PSQI**		
	Mean diff.	95.00% CI of diff.	p-value	Mean diff.	95.00% CI of diff.	p-value	Mean diff.	95.00% CI of diff.	p-value	Mean diff.	95.00% CI of diff.	p-value	Mean diff.	95.00% CI of diff.	p-value
**Baseline**															
Encapsulated probiotic vs Non-encapsulated probiotic	3.42	-3.03 to 9.87	0.2583	2.71	-1.56 to 6.98	0.1827	0.71	-1.88 to 3.30	0.5517	3.54	-1.62 to 8.70	0.1587	1.83	-1.57 to 5.23	0.2601
Encapsulated probiotic vs Placebo	3.58	-2.96 to 10.13	0.2488	2.96	-1.34 to 7.26	0.1535	0.63	-2.02 to 3.27	0.6118	2.63	-2.64 to 7.89	0.2988	0.11	-3.54 to 3.75	0.9504
Non-encapsulated probiotic vs Placebo	0.17	-2.75 to 3.08	0.9063	0.25	-1.50 to 2.00	0.7694	-0.08	-1.50 to 1.33	0.9036	-0.92	-4.34 to 2.50	0.5835	-1.72	-4.25 to 0.81	0.1710
**Week 3**															
Encapsulated probiotic vs Non-encapsulated probiotic	4.00	-3.43 to 11.43	0.2493	3.33	-1.34 to 8.00	0.1390	0.67	-2.40 to 3.73	0.6313	3.04	-0.67 to 6.75	0.1001	1.49	-1.69 to 4.68	0.3332
Encapsulated probiotic vs Placebo	5.67	-1.81 to 13.14	0.1195	4.33	-0.34 to 9.01	0.0652	1.33	-1.74 to 4.41	0.3502	2.13	-1.79 to 6.04	0.2658	0.61	-2.66 to 3.87	0.6978
Non-encapsulated probiotic vs Placebo	1.67	-0.99 to 4.32	0.2054	1.00	-0.71 to 2.71	0.2383	0.67	-0.56 to 1.89	0.2702	-0.92	-4.14 to 2.30	0.5603	-0.89	-3.70 to 1.93	0.5205
**Week 6**															
Encapsulated probiotic vs Non-encapsulated probiotic	5.71	-1.64 to 13.05	0.1108	4.71	0.21 to 9.21	0.0424	1.00	-2.52 to 4.52	0.5323	3.13	-1.85 to 8.10	0.1921	2.04	-1.08 to 5.15	0.1789
Encapsulated probiotic vs Placebo	5.63	-1.87 to 13.12	0.1246	4.21	-0.36 to 8.77	0.0667	1.42	-2.16 to 4.99	0.3955	3.63	-1.50 to 8.75	0.1490	0.94	-2.45 to 4.33	0.5624
Non-encapsulated probiotic vs Placebo	-0.08	-3.28 to 3.11	0.9569	-0.50	-2.28 to 1.28	0.5642	0.42	-1.12 to 1.96	0.5787	0.50	-2.62 to 3.62	0.7420	-1.10	-3.64 to 1.44	0.3793
**Week 10-12**															
Encapsulated probiotic vs Non-encapsulated probiotic	2.79	-3.75 to 9.33	0.3592	2.63	-1.73 to 6.98	0.2058	0.17	-2.64 to 2.97	0.8969	3.13	-1.91 to 8.16	0.2040	2.58	-0.512 to 5.67	0.0945
Encapsulated probiotic vs Placebo	3.29	-3.57 to 10.15	0.3156	2.88	-1.57 to 7.32	0.1808	0.42	-2.60 to 3.44	0.7708	2.42	-2.59 to 7.42	0.3178	1.40	-1.92 to 4.72	0.3834
Non-encapsulated probiotic vs Placebo	0.50	-3.44 to 4.44	0.7930	0.25	-2.05 to 2.55	0.8233	0.25	-1.73 to 2.23	0.7940	-0.71	-4.75 to 3.34	0.7198	-1.18	-3.80 to 1.44	0.3600
**Encapsulated probiotic**															
Baseline vs Week 3	-0.75	-2.28 to 0.78	0.2849	-0.63	-1.62 to 0.37	0.1803	-0.13	-0.95 to 0.70	0.7318	2.00	-1.13 to 5.13	0.1743	-0.50	-1.60 to 0.59	0.3159
Baseline vs Week 6	-1.63	-3.03 to -0.22	0.0294	-1.00	-2.10 to 0.09	0.0676	-0.63	-1.62 to 0.37	0.1803	1.25	-1.81 to 4.31	0.3656	-0.63	-2.46 to 1.21	0.4481

Baseline vs Week 10-12	0.38	-0.39 to 1.14	0.2849	0.25	-0.49 to 0.99	0.4512	0.13	-0.41 to 0.66	0.5983	1.50	0.02 to 2.98	0.0479	-0.75	-2.07 to 0.57	0.2216
Week 3 vs Week 6	-0.88	-2.39 to 0.64	0.2133	-0.38	-1.26 to 0.51	0.3506	-0.50	-1.60 to 0.59	0.3159	-0.75	-4.06 to 2.56	0.6083	-0.13	-1.34 to 1.09	0.8153
Week 3 vs Week 10-12	1.13	-0.57 to 2.82	0.1612	0.88	-0.07 to 1.82	0.0639	0.25	-0.91 to 1.41	0.6263	-0.50	-3.22 to 2.22	0.6767	-0.25	-1.90 to 1.40	0.7318
Week 6 vs Week 10-12	2.00	0.66 to 3.34	0.0096	1.25	0.18 to 2.32	0.0282	0.75	-0.12 to 1.62	0.0796	0.25	-2.93 to 3.43	0.8580	-0.13	-2.42 to 2.17	0.9013
**Non-encapsulated probiotic**															
Baseline vs Week 3	-0.17	-1.10 to 0.77	0.7014	0.00	-0.47 to 0.47	>0.9999	-0.17	-0.97 to 0.64	0.6576	1.50	-0.27 to 3.27	0.0884	-0.83	-1.80 to 0.14	0.0854
Baseline vs Week 6	0.67	-0.28 to 1.62	0.1513	1.00	0.19 to 1.81	0.0204	-0.33	-0.83 to 0.16	0.1661	0.83	-0.72 to 2.39	0.2623	-0.42	-1.21 to 0.37	0.2691
Baseline vs Week 10-12	-0.25	-1.81 to 1.31	0.7308	0.17	-1.10 to 1.43	0.7774	-0.42	-1.29 to 0.46	0.3177	1.08	-0.72 to 2.89	0.2137	0.00	-0.47 to 0.47	>0.9999
Week 3 vs Week 6	0.83	-0.31 to 1.98	0.1372	1.00	0.19 to 1.81	0.0204	-0.17	-0.87 to 0.54	0.6147	-0.67	-2.32 to 0.99	0.3944	0.42	-0.46 to 1.29	0.3177
Week 3 vs Week 10-12	-0.08	-1.45 to 1.28	0.8957	0.17	-1.13 to 1.46	0.7822	-0.25	-1.07 to 0.57	0.5152	-0.42	-2.36 to 1.53	0.6462	0.83	-0.17 to 1.84	0.0960
Week 6 vs Week 10-12	-0.92	-2.05 to 0.22	0.1023	-0.83	-1.77 to 0.10	0.0748	-0.08	-0.77 to 0.61	0.7949	0.25	-1.49 to 1.99	0.7574	0.42	-0.42 to 1.25	0.2945
**Placebo**															
Baseline vs Week 3	1.33	-0.003 to 2.67	0.0504	0.75	-0.07 to 1.57	0.0688	0.58	-0.37 to 1.54	0.2064	1.50	-0.68 to 3.68	0.1574	0.00	-0.78 to 0.78	>0.9999
Baseline vs Week 6	0.42	-1.22 to 2.05	0.5863	0.25	-0.87 to 1.37	0.6332	0.17	-0.59 to 0.92	0.6380	2.25	-0.19 to 4.69	0.0673	0.21	-1.01 to 1.43	0.7148
Baseline vs Week 10-12	0.08	-2.35 to 2.51	0.9412	0.17	-1.36 to 1.70	0.8148	-0.08	-1.25 to 1.08	0.8776	1.29	-1.63 to 4.21	0.3511	0.54	-0.82 to 1.91	0.4009
Week 3 vs Week 6	-0.92	-2.44 to 0.60	0.2112	-0.50	-1.42 to 0.42	0.2562	-0.42	-1.21 to 0.37	0.2691	0.75	-1.19 to 2.69	0.4123	0.21	-0.50 to 0.92	0.5315
Week 3 vs Week 10-12	-1.25	-3.69 to 1.19	0.2835	-0.58	-1.65 to 0.48	0.2534	-0.67	-2.18 to 0.85	0.3541	-0.21	-3.01 to 2.59	0.8729	0.54	-0.35 to 1.44	0.2088
Week 6 vs Week 10-12	-0.33	-1.94 to 1.28	0.6576	-0.08	-1.08 to 0.91	0.8569	-0.25	-1.19 to 0.69	0.5715	-0.96	-2.72 to 0.80	0.2555	0.33	-0.58 to 1.25	0.4382

Supplementarty Table 2. *Resting state functional connectivity at discontinuation follow-up (week 10-12) versus baseline. Significant time*group interactions; all voxel threshold p<0.001 uncorrected and cluster threshold p<0.05 with exact p-value as indicated (hypothesis-driven seed-to-voxel analysis (two-sided), regions annotation based on Harvard-Oxford atlas). Table only reports significant alterations, yet all 21 a priori defined regions of interest were tested as seeds. None of those effects remained significant, when additional multiplicity correction is applied for the number of seeds tested, comparisons of two pairs of timepoints and of three intervention groups (cluster size p<0.0004 FDR-corrected).*

Group comparison	*A priori* selected seed region	Target cluster size [number of voxels]	Target cluster-size p-FDR corrected	Target cluster peak coordinate [xx yy zz]	Target cluster coverage (order of anatomical regions based on proportion of coverage from largest to smallest)	Intervention-related alterations of connectivity [T]	Intervention-related alterations of connectivity##
**Placebo vs Encapsulated probiotic**	Superior Frontal gyrus Left#	135	0.0455	-48 +4 -18	Temporal Pole Left, Superior Temporal Gyrus anterior division Left, Middle Temporal Gyrus anterior division Left, Planum Polare Left	-6.66	- anti-correlation changed to correlation upon intake and subsequent discontinuation of the encapsulated probiotic<br>- correlation alteration was neglectable upon intake and subsequent

								discontinuation of the placebo
**Placebo vs Non-encapsulated probiotic**	Hippocampus Left[#]	233	0.0026	-44 +38 +6	Frontal Pole Left, Inferior Frontal Gyrus pars triangularis Left[#]	+6.17	-<br>-	anti-correlation decreased upon intake and subsequent discontinuation of the non-encapsulated probiotic<br>correlation decreased upon intake and subsequent discontinuation of the placebo
		105	0.0432	+36 -84 +26	Lateral Occipital Cortex superior division Right	-6.02	-	anti-correlation increased upon intake and subsequent discontinuation of

							the non-encapsulated probiotic<br>- anti-correlation changed to correlation upon intake and subsequent discontinuation of the placebo
	Amygdala Left#	197	0.0059	-38 +44 -4	Frontal Pole Left, Inferior Frontal Gyrus pars triangularis Left#, Frontal Orbital Cortex Left	+5.75	- anti-correlation decreased upon intake and subsequent discontinuation of the non-encapsulated probiotic<br>- correlation decreased upon intake and subsequent

							discontinuation of the placebo
**Encapsulated probiotic vs Non-encapsulated probiotic**	Middle Frontal Gyrus Right#	189	0.0141	-24 -32 -28	Cerebellum Left, Brainstem, Parahippocampal Gyrus posterior division Left, Temporal Fusiform Cortex posterior division Left	+10.25	- anti-correlation increased upon intake and subsequent discontinuation of the non-encapsulated probiotic<br>- anti-correlation changed to correlation upon intake and subsequent discontinuation of the encapsulated probiotic

#*a priori* defined region of interest.

##Synchronised brain activity patterns are described as correlations (e.g. two brain regions showing the same activity pattern) and anti-correlations (e.g. two brain regions showing opposite activity patterns), direction of correlation was interpreted as changed (from correlation to anti-correlation or vice versa), when error bar (based on standard error of the mean across individual voxels) did not cross zero

Supplementary Table 3. *Resting state functional connectivity at discontinuation follow-up (week 10-12) versus intervention end (week 6). Significant time*group interactions; all voxel threshold p<0.001 uncorrected and cluster threshold p<0.05 with exact p-value as indicated (hypothesis-driven seed-to-voxel analysis (two-sided), regions annotation based on Harvard-Oxford atlas). Table only reports significant alterations, yet all 21 a priori defined regions of interest were tested as seeds. In italics highlighted alterations remain significant, when additional multiplicity correction is applied for the number of seeds tested, comparisons of two pairs of timepoints and of three intervention groups (cluster size p<0.0004 FDR-corrected).*

**Group comparison**	***A priori* selected seed region**	**Target cluster size [number of voxels]**	**Target cluster-size p-FDR corrected**	**Target cluster peak coordinate [xx yy zz]**	**Target cluster coverage (order of anatomical regions based on proportion of coverage from largest to smallest)**	**Intervention-related alterations of connectivity [T]**	**Intervention-related alterations of connectivity[##] [description]**
**Placebo vs Encapsulated probiotic**	--- none ---						
**Placebo vs Non-encapsulated probiotic**	Middle Frontal Gyrus Right[#]	159	0.0298	+6 -76 -12	Lingual Gyrus Right, Vermis 6, Cerebellum Right	-7.22	- correlation changed to anti-correlation upon discontinuation of the non-encapsulated probiotic<br>- anti-correlation changed to correlation upon

							discontinuation of the placebo
	Inferior Frontal Gyrus pars opercularis Left#	253	0.0016	+2 -82 +10	Supracalcarine Cortex Right & Left, Occipital Pole Right & Left, Intracalcarine Cortex Right & Left, Cuneal Cortex Right & Left	+5.18	- correlation increased upon discontinuation of the non-encapsulated probiotic<br>- correlation changed to anti-correlation upon discontinuation of the placebo
	Precuneus#	148	0.0182	-2 -44 +30	Cingulate Gyrus posterior division#	-7.04	- correlation decreased upon discontinuation of the non-encapsulated probiotic<br>- correlation increased upon discontinuation of the placebo
**Encapsulated probiotic vs**	*Superior Parietal Lobule Left#*	*379*	*0.0001*	*+20 -96 +30*	*Occipital Pole Right, Lateral Occipital Cortex superior division Right, Cuneal Cortex Right, Intracalcarine Cortex Right, Supracalcarine Cortex Right*	*+7.38*	- *correlation decreased upon discontinuation of the non-encapsulated probiotic*

**Non-encapsulated probiotic**							- *correlation increased upon discontinuation of the encapsulated probiotic*
	Supramarginal Gyrus posterior division Right#	145	0.0270	+60 -22 +36	Supramarginal Gyrus anterior division Right#, Postcentral Gyrus Right	-6.41	- correlation increased upon discontinuation of the non-encapsulated probiotic<br>- correlation decreased upon discontinuation of the encapsulated probiotic

#*a priori* defined region of interest.

##Synchronised brain activity patterns are described as correlations (e.g. two brain regions showing the same activity pattern) and anti-correlations (e.g. two brain regions showing opposite activity patterns), direction of correlation was interpreted as changed (from correlation to anti-correlation or vice versa), when error bar (based on standard error of the mean across individual voxels) did not cross zero
