# Supplementary material for: Probiotic effects on brain health are both transient and sustained for several weeks after discontinuation: a randomised controlled trial
Source: Commun Med (Lond). 2026 Jul 28;6:418. doi: 10.1038/s43856-026-01800-6 (PMC13415881; doi:10.1038/s43856-026-01800-6)
Supplement: Supplementary file 1 — Transparent Peer Review file [file 43856_2026_1800_MOESM1_ESM.pdf]

nature portfolio

Peer Review File

Probiotic effects on brain health are both transient and sustained for several weeks after discontinuation: a randomised controlled trial

Corresponding Author: Dr Julia Rode

This file contains all reviewer reports in order by version, followed by all author rebuttals in order by version.

Version 0:

Reviewer comments:

Reviewer #1

(Remarks to the Author)
REVIEW: Probiotic effects on brain health are both transient and sustained for several weeks after discontinuation of daily administration
Understanding the health impacts of probiotic supplementation, and potential sustained effects, remain important avenues for investigation. This manuscript reports on exploratory follow-up analyses of older adults 4-6 weeks following participation in a RCT probiotic intervention. Immediate post-intervention effects have been published separately, investigating mood and brain imaging (MRI) outcomes. A smaller sub-group of overall trial participants (approximately one-third) are included in this manuscript. The manuscript reports self-report mood measures of anxiety and stress differed significantly between the discontinuation follow-up and immediate intervention end as well as mid-intervention, but not baseline, indicating that scores return to baseline (though note the main trial did not report significant effects on these in primary analyses). Structural brain changes reported in primary post-intervention analyses were not reported to persist, while functional changes were reported to have a mixed pattern. Overall, the manuscript tackles an important area of study – exploring sustained impact of intervention with probiotics, although there are substantial challenges in identifying robust trends in the sub-set of the overall trial.

1. Reporting outcomes of conventional statistical tests for mood outcomes, only p-values seem to be reported, without summary stats of measures, test values and degrees of freedom. In such small samples, with scores in a limited range, and with potential for floor effects in mood disturbance symptom severity, would nonparametric analyses be more appropriate (and consistent with the reporting of median and IQR in figures)? Indeed, the reported mood effects in this subset, that were absent in the main trial analyses, highlight the potential issues with this exploratory analyses in such a small sample.
2. While the manuscript is framed as exploratory, a potential concern is the number of statistical comparisons: Important efforts to account for the large number of tests have been made – however, there seems considerably more repeated tests in the resting state analyses – for example, contrasting each intervention group pair, and pairs of timepoints – raising the risk of false positives. The heterogenous pattern of interaction effects points to this potential for spurious findings – potentially driven by shifts in placebo of at least equal magnitude. These trends being largely divergent from those observed in the main trial report also highlight this threat. Have there been further efforts to adjust for these tests (not just across the 21 ROIs)? The main published trial analyses for rs-fMRI seemed to begin with a requirement for significant overall group x time F-test before conducting subsequent pairs of interaction analyses (and then use correction for posthoc tests) – was this also conducted here? This first overall group x time analysis would also seem to be consistent with the structural brain outcome analyses?

3. Line 358: Reporting non-significant effects observed in the larger trial is potentially misrepresenting the findings?
4. When summarising regional effects in Resting State Connectivity (from line 278), referring to anatomical regions seems more precise than assigning functional labels to the observed effects.
5. Line 176: Statistical analyses: clarify was it the Group x Time interaction that must be significant in order to evaluate posthoc contrasts?
6. Line 196: In preprocessing of the resting state data, was the study-specific anatomical template generated as part of the DARTEL pipeline used to improve the spatial registration to MNI space?
7. Line 214: If this is using parametric methods (please specify), then referring to the cluster corrected FDR q<.05 with p<.001 cluster defining threshold arguably should not be referred to as the accepted standard and overlooks considerable

complexity and debate.
8. Line 221: capitalise DARTEL, and cite method?

Reviewer #2

(Remarks to the Author)
Hutchinson et al. examine transient and sustained effects of a probiotic supplement after discontinuation on brain health in 32 community-dwelling older adults. The research question is highly relevant, as it remains largely unclear to what extent probiotic-induced effects persist once supplementation has ended. The study addresses an important and underexplored aspect of probiotic research, particularly in an aging population.
I believe the manuscript has strong potential. However, given the relatively high number of comparisons, some sections of the manuscript are challenging to follow. Streamlining the narrative and making certain points more explicit may substantially enhance readability. I hope my comments may help the authors to improve the manuscript.

- Role of the VBM analysis: The VBM analysis is an interesting addition; however, its role in the overall study rationale could be made clearer. As it is not introduced in the Introduction, readers may benefit from a brief explanation of why VBM was included and how it relates to findings from the previous publication. Explicitly naming the key results from the earlier analysis that motivated its inclusion would strengthen coherence.
- Reporting of resting-state fMRI results: The reporting of the resting-state results could be slightly more explicit. In particular, it would be helpful to clearly state that, in the follow-up vs. baseline comparison, none of the effects remain significant after correction for multiple comparisons (Table 2).
- Differences between probiotic interventions: The observed differences between the two probiotic interventions (Figure 5) are intriguing and would benefit from a discussion of potential underlying mechanisms. More generally, the resting-state fMRI findings could be discussed in greater depth. At present, the Discussion places stronger emphasis on the distinction between transient vs sustained effects, while the interpretation of the specific fMRI results themselves could be further elaborated.
- Link between imaging and psychological measures: Given the modest sample size, it is reasonable that the authors refrain from directly linking rsfMRI findings with psychological measures. Nonetheless, it may be valuable to explicitly highlight this as an important avenue for future research, as correlating behavioral or psychological outcomes with imaging data would greatly enhance the interpretability and functional relevance of the findings.

Points requiring clarification
A few statements in the Discussion section would benefit from clarification to avoid potential confusion:
- “Interestingly, this connectivity alteration is one of two in the herein presented work, and the only one in the reporting of the immediate intervention effects, that remain statistically significant even after additional more strict multiplicity correction for the number of seeds tested.”
My understanding is that the SPL connectivity difference between encapsulated and non-encapsulated probiotics is not the only effect that remains significant after additional correction, as the IFG effect appears to do so as well. Could you please clarify?
- “We further validated our fMRI results, by confirming that the default mode network could be picked up reliantly, despite the small number of subjects per group.”
The relevance of the default mode network (DMN) to the overall manuscript is not entirely clear. As the DMN is only mentioned once in relation to the SFG, it may be helpful to clarify why successful DMN identification is important for validating or interpreting the present results.
- “The interpretation of the herein reported results is limited by a comparison to previously assessed immediate intervention effects in a larger study population.”
This sentence could be made more precise. It appears that only analyses that showed significant effects in the previous study were repeated but the then investigated seed contain more than the previously reported significant effects.

Reviewer #3

(Remarks to the Author)
The manuscript highlights an interesting gap in the current literature. However, the following major recommendations are needed to improve the manuscript.
1-The sample size, including the end of the follow-up period, is too small to come to a definitive conclusion regarding the neuroimaging correlates of the postintervention effects of the probiotic agent. This should be discussed and acknowledged as a limitation in the relevant section of the manuscript
2-The raw scores of each period, including a detailed description of the altered mean scores between baseline intervention and postintervention end points, should be defined. Any group difference? (Repeated measures regarding pre- and post-periods?)
3-Are there any correlations between altered neuroimaging data and depression and anxiety scores? Correlation and regression analysis is needed in that context
4-The authors should also discuss their findings indicating a difference between non-encapsulated probiotics and placebo in the light of current literature
5-Why the authors did not apply roi to roi analysis?

Version 1:

Reviewer comments:

Reviewer #1

(Remarks to the Author)
The authors have undertaken several changes to address reviewer comments.

My main comment remains the justification for a parametric approach to mood analyses, as the inspection of Q-Q plots is difficult when such plots are sample-size dependent. In such small samples, with scores in a limited range (zero-bounded) – and evidence of floor effects, and potential baseline differences between groups in symptom severity, would nonparametric analyses be more appropriate? This is also consistent with the reporting of median and IQR in figures.

Reviewer #2

(Remarks to the Author)
The authors have substantially improved the manuscript, and I appreciate the extensive revisions. I only have a few remaining minor comments:
1. VBM analysis: The rationale for the VBM analysis still remains insufficiently clear to me. Is the aim to (a) replicate the null findings from the first paper (i.e., the previously reported non-significant changes) or (b) examine the previously observed subtle changes that reached significance only before correction for multiple comparisons? If the latter, it would be helpful to indicate which regions showed these uncorrected subtle changes in the first paper.
In addition, lines 285–286 state that "Structural brain changes do not persist." This statement seems misleading.
2. Reporting of statistics: I recommend reporting ANOVA results according to APA guidelines (i.e., F statistics first, followed by the p-value).
3. Discussion: In the Results section, the authors focus on the findings that remain significant after the additional correction for multiple comparisons. However, the Discussion addresses non-significant findings extensively (e.g., lines 487–497). I recommend clarifying more explicitly which effects remain significant and which are not to prevent confusion for the reader.

Reviewer #3

(Remarks to the Author)
Based on my review of Figure 3, I am not convinced there is a significant therapeutic effect between the baseline and the intervention period. Please provide the raw scores in a table, including the statistical values that show: a) whether there is a significant benefit; b) if this therapeutic effect is maintained; and c) whether these proposed clinical changes are linked or interact with connectivity changes (using partial correlation or regression analysis).

Version 2:

Reviewer comments:

Reviewer #1

(Remarks to the Author)
The authors provide a detailed response and conduct additional sensitivity analyses, for which I am grateful. The inclusion of mixed-effects models and non-parametric tests is helpful, and the consistency of findings across approaches is reassuring.
I would note, however, that while ANOVA is often described as robust to moderate deviations from normality, that robustness is less well assured under the present combination of conditions (small n, bounded and skewed distributions, and floor effects), particularly for interaction terms.
The reported reduction in anxiety and HADS total scores from 6 weeks to follow-up appears, in absolute terms, to reflect a return to baseline (pre-treatment) levels. As no summary statistics are provided for each timepoint (beyond boxplots and supplementary difference tables), this pattern is somewhat difficult to fully evaluate. However, visual inspection of the boxplots suggests a non-monotonic trajectory, with scores tending to increase during the intervention period (baseline to week 6) and subsequently decline toward baseline at follow-up. The distributions also appear bounded with evidence of floor effects and substantial overlap between groups, with within-group variability exceeding between-group differences. Clarification of these temporal trends would be helpful, as the current presentation may suggest that anxiety increases during the intervention and then returns toward baseline levels in the follow-up period.
It would strengthen the manuscript to more explicitly acknowledge the limitations of parametric inference in this setting, to discuss how floor effects may influence estimation and interpretation of effects, and to temper the interpretation of factorial/interaction results accordingly—particularly in light of the broader trajectory of mood symptoms across the intervention period.

Reviewer #2

(Remarks to the Author)

The authors have satisfactorily addressed my concerns.

Version 3:

Reviewer comments:

Reviewer #1

(Remarks to the Author)
The authors have satisfactorily addressed my comments.

Open Access This Peer Review File is licensed under a Creative Commons Attribution 4.0 International License, which permits use, sharing, adaptation, distribution and reproduction in any medium or format, as long as you give appropriate credit to the original author(s) and the source, provide a link to the Creative Commons license, and indicate if changes were made.
In cases where reviewers are anonymous, credit should be given to 'Anonymous Referee' and the source.
The images or other third party material in this Peer Review File are included in the article's Creative Commons license, unless indicated otherwise in a credit line to the material. If material is not included in the article's Creative Commons license and your intended use is not permitted by statutory regulation or exceeds the permitted use, you will need to obtain permission directly from the copyright holder.
To view a copy of this license, visit https://creativecommons.org/licenses/by/4.0/

Chief Editor
Communications Medicine

Response to reviewer comments

To the Chief Editor,

On behalf of our co-authors, we would like to thank you for reviewing our manuscript titled “Probiotic effects on brain health are both transient and sustained for several weeks after discontinuation”. We thoroughly appreciate the reviewers insightful and beneficial comments. We have addressed the comments as discussed below, and we have indicated any changes in the manuscript (markup file).

Reviewer #1

> *REVIEW: Probiotic effects on brain health are both transient and sustained for several weeks after discontinuation of daily administration*
>
> *Understanding the health impacts of probiotic supplementation, and potential sustained effects, remain important avenues for investigation. This manuscript reports on exploratory follow-up analyses of older adults 4-6 weeks following participation in a RCT probiotic intervention. Immediate post-intervention effects have been published separately, investigating mood and brain imaging (MRI) outcomes. A smaller sub-group of overall trial participants (approximately one-third) are included in this manuscript. The manuscript reports self-report mood measures of anxiety and stress differed significantly between the discontinuation follow-up and immediate intervention end as well as mid-intervention, but not baseline, indicating that scores return to baseline (though note the main trial did not report significant effects on these in primary analyses). Structural brain changes reported in primary post-intervention analyses were not reported to persist, while functional changes were reported to have a mixed pattern. Overall, the manuscript tackles an important area of study – exploring sustained impact of intervention with probiotics, although there are substantial challenges in identifying robust trends in the sub-set of the overall trial.*

We are very pleased that the reviewer agrees that this is a very relevant and significant area in the fields of both probiotics and brain imaging. We also deeply appreciate the comments and suggestions to strengthen our manuscript.

1. *Reporting outcomes of conventional statistical tests for mood outcomes, only p-values seem to be reported, without summary stats of measures, test values and degrees of freedom. In such small samples, with scores in a limited range, and with potential for floor effects in mood disturbance symptom severity, would nonparametric analyses be more appropriate (and consistent with the reporting of median and IQR in figures)? Indeed, the reported mood effects in this subset, that were absent in the main trial analyses, highlight the potential issues with this exploratory analyses in such a small sample.*

We would like to thank the reviewer for suggesting additional reporting of performed statistical tests. We have now updated the results section to include F-values, t-

statistics and degrees of freedom. In order to determine whether to utilise parametric vs non-parametric analyses for the self-rated questionnaires, we inspected the data via QQ plots and determined that parametric analyses were appropriate for this data set, as stated in the methods section (lines 217-219). In response to a formatting requirement posted by the editorial office, we have now reported boxplots in the figures. In response to other comments from the reviewers, a more conservative statistical approach has been employed throughout the manuscript. Thus, only HADS Total Score and HADS Anxiety Subscore showed significant time*group interaction effects (lines 320-332). This is quite similar to the main study in which HADS Anxiety Subscore was significant, which is a subscore of the total score.

2. *While the manuscript is framed as exploratory, a potential concern is the number of statistical comparisons: Important efforts to account for the large number of tests have been made – however, there seems considerably more repeated tests in the resting state analyses – for example, contrasting each intervention group pair, and pairs of timepoints – raising the risk of false positives. The heterogenous pattern of interaction effects points to this potential for spurious findings – potentially driven by shifts in placebo of at least equal magnitude. These trends being largely divergent from those observed in the main trial report also highlight this threat. Have there been further efforts to adjust for these tests (not just across the 21 ROIs)? The main published trial analyses for rs-fMRI seemed to begin with a requirement for significant overall group x time F-test before conducting subsequent pairs of interaction analyses (and then use correction for posthoc tests) – was this also conducted here? This first overall group x time analysis would also seem to be consistent with the structural brain outcome analyses?*

As stated above, we appreciate the reviewer's insight and have now updated the manuscript to include more conservative statistical approaches, including a Bonferroni correction for 21 seeds, 2 timepoint pair, 3 group pairs instead of the former correction with for 21 seeds only.

While the additional multiplicity correction was performed within SBC and RRC analyses sets, respectively, the significant functional connectivity alteration (with SPL seed) would still remain significant even when applying additional correction across all rsfMRI analyses (i.e. 21 seeds, 3 analyses versions, 2 timepoint pair, 3 group pairs) which would result in Bonferroni-corrected $p<0.00013$. Our approach of reporting of p-values, allows the reader to perform Bonferroni correction at any level and to draw their own conclusions, as also pointed out in lines 618-621. This aligns well with the exploratory and hypothesis-generating character of this study.

Hence, lines 267-273 in the methods section have been included/updated.

Only results that remain significant after additional correction are now presented in the main text, current figure 4, current table 2. Captions of the respective tables/figures and lines 367-406 have been updated. Former tables 2 and 3 have been moved to the Supplementary Information, Also, former figure 4 has been removed.

F-tests have now been performed, which is now clarified in line 259, and its result for the altered connectivity of the SPL seed is reported in lines 398-402.

3. *Line 358: Reporting non-significant effects observed in the larger trial is potentially misrepresenting the findings?*

We have now removed the non-significant effects observed in the larger trial (lines 472-473).

4. *When summarising regional effects in Resting State Connectivity (from line 278), referring to anatomical regions seems more precise than assigning functional labels to the observed effects.*

We agree with the reviewers that it is more precise to use anatomical regions rather than functional labels. We have thus updated the results section accordingly (lines 371-402) and moved functional labels to the discussion (lines 519-535).

5. *Line 176: Statistical analyses: clarify was it the Group x Time interaction that must be significant in order to evaluate posthoc contrasts?*

We have now clarified that the time*group interaction must be significant in order to evaluate posthoc contrasts (new line 221). Consequently, also results reporting (lines 334-337) and discussion (lines 428-480, and 650) was updated.

6. *Line 196: In preprocessing of the resting state data, was the study-specific anatomical template generated as part of the DARTEL pipeline used to improve the spatial registration to MNI space?*

As stated and now clarified in lines 243-245, the preprocessing was conducted using the default pipeline implemented in CONN with spatial normalisation performed using the standard segmentation approach to MNI space. A study-specific anatomical template generated via DARTEL was not used for the rsfMRI analyses, and solely for the VBM analyses.

7. *Line 214: If this is using parametric methods (please specify), then referring to the cluster corrected FDR q<.05 with p<.001 cluster defining threshold arguably should not be referred to as the accepted standard and overlooks considerable complexity and debate.*

We agree with the reviewer that the phrase "accepted standard in the field" may be misleading; thus, we have removed the phrase (lines 266-267). We have also specified that we utilised parametric methods for rsfMRI analyses (line 261).

8. *Line 221: capitalise DARTEL, and cite method?*

DARTEL is now capitalised and the original DARTEL paper is cited (line 285).

Reviewer #2

Hutchinson et al. examine transient and sustained effects of a probiotic supplement after discontinuation on brain health in 32 community-dwelling older adults. The research question is highly relevant, as it remains largely unclear to what extent probiotic-induced effects persist once supplementation has ended. The study addresses an important and underexplored aspect of probiotic research, particularly in an aging population.

I believe the manuscript has strong potential. However, given the relatively high number of comparisons, some sections of the manuscript are challenging to follow. Streamlining the narrative and making certain points more explicit may substantially enhance readability. I hope my comments may help the authors to improve the manuscript.

We would like to thank the reviewer for the comprehensive review of our manuscript. We are pleased to hear that the reviewer appreciates the novelty and potential of our study, and we deeply appreciate the insightful comments to improve our manuscript.

- Role of the VBM analysis: The VBM analysis is an interesting addition; however, its role in the overall study rationale could be made clearer. As it is not introduced in the Introduction, readers may benefit from a brief explanation of why VBM was included and how it relates to findings from the previous publication. Explicitly naming the key results from the earlier analysis that motivated its inclusion would strengthen coherence.

We agree with the reviewer that more explanation about VBM and its relevance to the current manuscript is helpful for our readers. Thus, in the beginning of the results section "Structural brain assessment", we have now included a sentence to indicate our rational for including VBM based on the findings from the 2025 publication (lines 454-456).

- Reporting of resting-state fMRI results: The reporting of the resting-state results could be slightly more explicit. In particular, it would be helpful to clearly state that, in the follow-up vs. baseline comparison, none of the effects remain significant after correction for multiple comparisons (Table 2).

To improve transparency, we have now updated the caption for former Table 2, which is now Supplementary Table 1 to clearly state that "none of those effects remained significant" after additional and conservative correction for multiple comparisons.

- Differences between probiotic interventions: The observed differences between the two probiotic interventions (Figure 5) are intriguing and would benefit from a discussion of potential underlying mechanisms. More generally, the resting-state fMRI findings could be discussed in greater depth. At present, the Discussion places stronger emphasis on the distinction between transient vs sustained effects, while the interpretation of the specific fMRI results themselves could be further elaborated.

We agree with the reviewer that it is quite intriguing that there are differences in functional connectivity between the two probiotic formulations. We have now included an updated discussion of potential underlying mechanisms that account for the differential effects of the two formulations on brain connectivity, highlighting the more detailed discussions on this topic in our 2025 publication (lines 536-542). Furthermore, we have also included more detail about the functional domains that are differentially impacted by the two probiotic formulations, noting that, interestingly, the differences we see in the current manuscript largely resemble what we observed at immediate intervention end reported in our 2025 publication (lines 519-535).

- Link between imaging and psychological measures: Given the modest sample size, it is reasonable that the authors refrain from directly linking rsfMRI findings

with psychological measures. Nonetheless, it may be valuable to explicitly highlight this as an important avenue for future research, as correlating behavioral or psychological outcomes with imaging data would greatly enhance the interpretability and functional relevance of the findings.

We agree that future studies with larger sample sizes should focus on linking brain imaging and questionnaire data. We have presented this suggestion now in the discussion (lines 656-657).

Points requiring clarification

A few statements in the Discussion section would benefit from clarification to avoid potential confusion:

- "Interestingly, this connectivity alteration is one of two in the herein presented work, and the only one in the reporting of the immediate intervention effects, that remain statistically significant even after additional more strict multiplicity correction for the number of seeds tested."

My understanding is that the SPL connectivity difference between encapsulated and non-encapsulated probiotics is not the only effect that remains significant after additional correction, as the IFG effect appears to do so as well. Could you please clarify?

With the more conservative statistical approach applied, only one of the two former significant functional connectivity alterations still remained significant. Hence, we have removed "one of two" to state now "the only one" in the present work as well as the "only one of the previously reported immediate intervention effects" that remained significant after strict multiplicity correction (lines 506-507). We believe that this should simultaneously improve clarity.

- "We further validated our fMRI results, by confirming that the default mode network could be picked up reliantly, despite the small number of subjects per group."

The relevance of the default mode network (DMN) to the overall manuscript is not entirely clear. As the DMN is only mentioned once in relation to the SFG, it may be helpful to clarify why successful DMN identification is important for validating or interpreting the present results.

We agree that the relevance of the DMN was not clear; thus, we have moved this to the methods section where we now highlight that we detected the DMN to assess the validity of our fMRI acquisition and results in our small sample size (lines 251-255).

- "The interpretation of the herein reported results is limited by a comparison to previously assessed immediate intervention effects in a larger study population."

This sentence could be made more precise. It appears that only analyses that showed significant effects in the previous study were repeated but the then investigated seed contain more than the previously reported significant effects.

Indeed, the analyses were not limited to outcomes significant at immediate intervention end. This has now been clarified in lines 639-614 which now reads "Generally, the analyses of discontinuation effects were restricted to psychological symptom scores as

well as structural and functional brain changes.”. We agree that even the sentence highlighted by the reviewer, may be misunderstood. Hence, we also clarified this now (lines 603-606).

Reviewer #3

The manuscript highlights an interesting gap in the current literature. However, the following major recommendations are needed to improve the manuscript.

We are very pleased that the reviewer believes our manuscript addresses a gap in the field, and we are thankful for insightful comments to improve our manuscript.

1-The sample size, including the end of the follow-up period, is too small to come to a definitive conclusion regarding the neuroimaging correlates of the postintervention effects of the probiotic agent. This should be discussed and acknowledged as a limitation in the relevant section of the manuscript

We agree that the sample size is too small for definitive conclusions. We have now updated the discussion to more strongly emphasise this point (lines 609).

2-The raw scores of each period, including a detailed description of the altered mean scores between baseline intervention and postintervention end points, should be defined. Any group difference? (Repeated measures regarding pre- and post-periods?)

As we have now clarified in the discussion (lines 603-608), we are not able to again analyse and report on the baseline vs postintervention results, as this was already reported in the previous manuscript. However, test statistics have now been included for HADS total, HADS A, PSS, and rsfMRI (lines 320-337, and 379-402).

3-Are there any correlations between altered neuroimaging data and depression and anxiety scores? Correlation and regression analysis is needed in that context

The sample size of the current study is too small to conduct such correlations, but we agree with the reviewer that this is a very important direction for future studies. We have noted this potential future direction in the discussion (lines 656-657).

4-The authors should also discuss their findings indicating a difference between non-encapsulated probiotics and placebo in the light of current literature

We have now included such comparison in the discussion lines 509-511 and 533-542.

5-Why the authors did not apply roi to roi analysis?

We agree with the reviewer that ROI-to-ROI analysis strengthens the manuscript. Thus, we have now completed this analysis for whole-brain and the 21 *a priori* regions of interest (lines 274-280 in the methods, lines 407-415 in the results section and lines 512-514 in the discussion).

Additional updates

Upon further review we choose to clarify the last column “intervention-related alterations of connectivity” in current Table 2 and Supplementary Tables 1 and 2, to increase readability.

The formatting requests posted by the editorial office have been addressed.

We hope that you find that we have sufficiently addressed the reviewers’ comments. Thank you for reviewing and considering this manuscript for publication. We look forward to your response.

Respectfully yours,

Dr Ashley N Hutchinson MSc PhD, Prof Robert J Brummer MD PhD, and Dr Julia Rode MSc PhD

Chief Editor
Communications Medicine

Response to reviewer comments

To the Chief Editor,

On behalf of our co-authors, we would again like to thank you for reviewing our manuscript titled “Probiotic effects on brain health are both transient and sustained for several weeks after discontinuation”. We thoroughly appreciate the reviewers insightful and beneficial comments. We have addressed the comments as discussed below, and we have indicated any changes in the manuscript (markup file).

Reviewers' comments:

Reviewer #1 (Remarks to the Author):

The authors have undertaken several changes to address reviewer comments.

My main comment remains the justification for a parametric approach to mood analyses, as the inspection of Q-Q plots is difficult when such plots are sample-size dependent. In such small samples, with scores in a limited range (zero-bounded) – and evidence of floor effects, and potential baseline differences between groups in symptom severity, would nonparametric analyses be more appropriate? This is also consistent with the reporting of median and IQR in figures.

We agree that various statistical approaches may be valid and that each may have its advantages and disadvantages. We had chosen to conduct two-way ANOVA analyses of the psychological symptom scores since this allows the full utilisation of our design, i.e. modelling of not only main but also interaction effects, which may also be lost in rank-based ANOVA. While non-parametric alternatives for factorial designs exist, many either do not fully accommodate interaction effects or repeated measures structures or require transformation of the data that complicates interpretation. We acknowledge that QQ plots of residuals are e.g. sample size dependent and that our visual inspection thereof may hence be limited. However, parametric methods such as ANOVA are typically robust to moderate deviations from normality, especially in balanced designs. Due to all of the above, we have now additionally conducted linear mixed-effects model analyses in SPSS, which are possibly even more robust to moderate deviations from normality. We have performed those analyses with and without modelling individual trajectories or steady correlation of measurements over time. We have now also conducted sensitivity analyses using non-parametric Friedman tests per group and Kruskal-Wallis tests for between-group differences. While these tests do not fully reproduce the factorial design structure either, they provide complementary assessments of within- and between-group differences. The results of the latter are

presented in Supplementary Information 1, which is referenced in lines 213-214. Both approaches yielded results consistent with the presented parametric analyses, supporting the robustness of effects under different distributional assumptions. Individual test statistics, including p-values, may differ slightly, not surprisingly considering the different test principles.

Baseline psychological symptom ratings did not differ between groups. We have added this information to lines 567-568. Furthermore, we have included this information in Supplementary Table 1, which is referred to lines 319-320; this information was requested by Reviewer 3.

While a two-way ANOVA works under the assumption of normally distributed residuals, the original data does not necessarily need to be normally distributed. Hence, the usage of a parametric test does not necessarily contradict presentation of original data as median and IQR. Their presentation acknowledges bounded distributions and floor effects. This is also in line with the editorial checklist that asks for presentation as boxplots (or individual data points which would result in messy, incomprehensible graphs in our case). A standard boxplot typically presents median, not mean, hence a change presenting mean may lead to confusion of the reader.

Taken together, we believe that the consistency of results across parametric, mixed-effects, and non-parametric approaches supports the robustness of our findings and justifies for our choices.

Reviewer #2 (Remarks to the Author):

The authors have substantially improved the manuscript, and I appreciate the extensive revisions. I only have a few remaining minor comments:

1. VBM analysis: The rationale for the VBM analysis still remains insufficiently clear to me. Is the aim to (a) replicate the null findings from the first paper (i.e., the previously reported non-significant changes) or (b) examine the previously observed subtle changes that reached significance only before correction for multiple comparisons? If the latter, it would be helpful to indicate which regions showed these uncorrected subtle changes in the first paper.

In addition, lines 285–286 state that "Structural brain changes do not persist." This statement seems misleading.

We are pleased to hear that the reviewer finds that our revisions have substantially improved the manuscript.

We clarified the exploratory character of our work; please refer to lines 384-389. We have removed the sentence “Structural brain changes do not persist.” now, current lines 417-418.

2. Reporting of statistics: I recommend reporting ANOVA results according to APA guidelines (i.e., F statistics first, followed by the p-value).

We have now changed the order, to always report the F statistics first, followed by the p-value.

3. Discussion: In the Results section, the authors focus on the findings that remain significant after the additional correction for multiple comparisons. However, the Discussion addresses non-significant findings extensively (e.g., lines 487–497). I recommend clarifying more explicitly which effects remain significant and which are not to prevent confusion for the reader.

We agree with the reviewer, and we have now tempered the discussion by introducing phrases such as “before additional multiplicity correction” or “non-significant” throughout.

Reviewer #3 (Remarks to the Author):

Based on my review of Figure 3, I am not convinced there is a significant therapeutic effect between the baseline and the intervention period. Please provide the raw scores in a table, including the statistical values that show: a) whether there is a significant benefit; b) if this therapeutic effect is maintained; and c) whether these proposed clinical changes are linked or interact with connectivity changes (using partial correlation or regression analysis).

While the immediate intervention effects are reported in our previous publication Rode&Hutchinson et al. 2025, the herein presented work is an exploratory analysis in a subset of circa one third of the original study population. We had chosen not to reassess immediate intervention effects, as this would have been a reanalysis, reinterpretation, and republication of the same data in an arbitrary subset of participants not meeting the pre-set study criteria. This limitation had already been discussed (see lines 541-544), and this argument has now been updated accordingly. However, we understand that it is important for the reader to be able to assess those immediate intervention effects, despite its limitations, also in this subset in order to reasonably judge the discontinuation effects. We have therefore included current Supplementary Table 1 with the mean differences, and 95% CI thereof, for comparisons between all timepoints and intervention groups of the data underlying Figure 3. Descriptive, not multiplicity corrected p-values, are presented since any correction would be arbitrary and not reflect the actual analyses. The reader may hence apply a correction of their choice. The

numerical source data of Figure 3 had already been provided during the previous revision (Supplementary Data File).

Furthermore, we have now conducted a multiple linear regression analysis; please refer to lines 250-252 in the methods section and lines 356-361 in the results section. This also led to a small update in the discussion, lines 587-588, which referred to the answer of reviewer comments in the previous revision.

We hope that you find that we have sufficiently addressed the reviewers' comments. Thank you for reviewing and considering this manuscript for publication. We look forward to your response.

Respectfully yours,

Dr Ashley N Hutchinson MSc PhD, Prof Robert J Brummer MD PhD, and Dr Julia Rode MSc PhD

Chief Editor
Communications Medicine

Response to reviewer comments

To the Chief Editor,

On behalf of our co-authors, we would again like to thank you for reviewing our manuscript titled “Probiotic effects on brain health are both transient and sustained for several weeks after discontinuation: a randomised controlled trial”. We thoroughly appreciate the reviewers insightful and beneficial comments. We have addressed the comments as discussed below, and we have indicated any changes in the manuscript (markup file).

Reviewers' comments:

Reviewer #1 (Remarks to the Author):

The authors provide a detailed response and conduct additional sensitivity analyses, for which I am grateful. The inclusion of mixed-effects models and non-parametric tests is helpful, and the consistency of findings across approaches is reassuring.

I would note, however, that while ANOVA is often described as robust to moderate deviations from normality, that robustness is less well assured under the present combination of conditions (small n, bounded and skewed distributions, and floor effects), particularly for interaction terms.

The reported reduction in anxiety and HADS total scores from 6 weeks to follow-up appears, in absolute terms, to reflect a return to baseline (pre-treatment) levels. As no summary statistics are provided for each timepoint (beyond boxplots and supplementary difference tables), this pattern is somewhat difficult to fully evaluate. However, visual inspection of the boxplots suggests a non-monotonic trajectory, with scores tending to increase during the intervention period (baseline to week 6) and subsequently decline toward baseline at follow-up. The distributions also appear bounded with evidence of floor effects and substantial overlap between groups, with within-group variability exceeding between-group differences. Clarification of these temporal trends would be helpful, as the current presentation may suggest that anxiety increases during the intervention and then returns toward baseline levels in the follow-up period.

It would strengthen the manuscript to more explicitly acknowledge the limitations of parametric inference in this setting, to discuss how floor effects may influence estimation and interpretation of effects, and to temper the interpretation of

factorial/interaction results accordingly—particularly in light of the broader trajectory of mood symptoms across the intervention period.

We thank the reviewer for the critical evaluation of our statistical approach. To clarify the remaining points, we have now included a description of the visually apparent pattern of HADS scores in the results section, please refer to lines 299-301 and 306-309. We have furthermore included a note of this in the respective discussion section (line 409). We acknowledge the limitations of our approach and have clarified this in lines 548-554 (incl move from 580-581) and 564-571. Summary statistics underlying all graphs are presented in the Supplementary Data File.

Reviewer #2 (Remarks to the Author):

The authors have satisfactorily addressed my concerns.

We are pleased to hear that reviewer 2 acknowledges our manuscript's improvements.

We hope that you find that we have sufficiently addressed the reviewers' comments. Thank you for reviewing and considering this manuscript for publication. We look forward to your response.

Respectfully yours,

Dr Ashley N Hutchinson MSc PhD, Prof Robert J Brummer MD PhD, and Dr Julia Rode MSc PhD
